# Supplementary material for: Autonomous self-healing supramolecular polymer transistors for skin electronics
Source: Nat Commun. 2024 Apr 23;15:3433. doi: 10.1038/s41467-024-47718-2 (PMC11039670; doi:10.1038/s41467-024-47718-2)
Supplement: Supplementary file 1 — Supplementary Information [file 41467_2024_47718_MOESM1_ESM.pdf]

Supplementary Information for

Autonomous Self-Healing Supramolecular Polymer Transistors for Skin Electronics

Ngoc Thanh Phuong Vo[1‡], Tae Uk Nam[1‡], Min Woo Jeong[1], Jun Su Kim[1], Kyu Ho Jung[1], Yeongjun Lee[2,3], Guorong Ma[4], Xiaodan Gu[4], Jeffrey B.-H. Tok[2], Tae Il Lee[5], Zhenan Bao[2]*, Jin Young Oh[1]**

[1]Department of Chemical Engineering (Integrated Engineering Program), Kyung Hee University, Yongin, Gyeonggi, 17104, Korea

[2]Department of Chemical Engineering, Stanford University, Stanford, CA 94305-5025, USA

[3]Department of Brain and Cognitive Sciences, KAIST, Daejeon, 34141, Korea

[4]School of Polymer Science and Engineering, University of Southern Mississippi, Hattiesburg, MS 39406, USA

[5]Department of Materials Science and Engineering, Gachon University, Seong-nam, Gyeonggi, 13120, Korea.

[‡]These authors have equal contribution to this work

*Corresponding author. Email: t2.lee77@gachon.ac.kr, jyoh@khu.ac.kr, zbao@stanford.edu

Table of Contents Summary

Supplementary Notes .... 1 - 4

Supplementary Note 1. Characteristics of the FETs 1

Supplementary Note 2. Calculation of elastic modulus ……………………….............. 2

Supplementary Note 3. Calculation method for dielectric constant ……………………. 3

Supplementary Note 4. Calculation of surface energies …………………………..……… 4

Supplementary Figures .... 5 - 83

Supplementary Fig. 1 to Fig. 79 .... 5 - 83

Supplementary Tables .... 84 - 85

Supplementary Table 1. Device geometry and dielectric capacitance under strain ………. 84

Supplementary Table 2. Comparison of device performance …………………….................. 85

Reference 86

Supplementary Notes

Supplementary Note 1. Characteristics of the FETs

The field effect mobility (μ_{FE}) is calculated in saturation regime by fitting the plot of the linear regime of square root of drain current ($\sqrt{I_D}$) versus gate voltage (V_G). The equation follows (S1):

$$\mu_{FE} = \frac{2L}{WC_i}\left(\frac{\partial\sqrt{I_D}}{\partial V_G}\right)^2 \tag{S1}$$

Where L is the length of the channel, W is width of the channel. C_i is capacitance per unit area of gate dielectric. The values of all parameters are shown at Table S1.

Supplementary Note 2. Calculation of elastic modulus using wrinkle forming method.

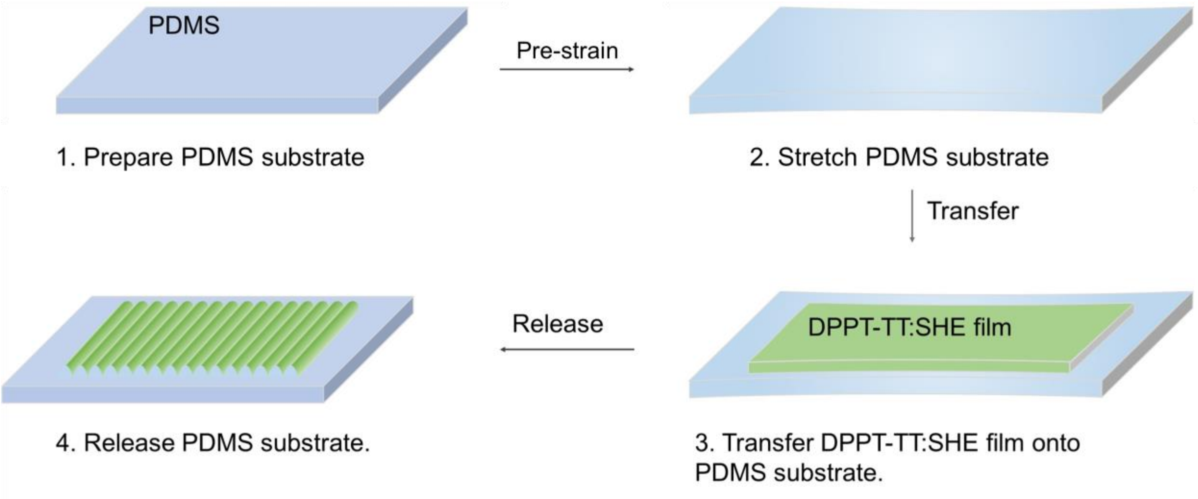

The elastic modulus of DPPT-TT:SHE films are measured using a buckling-based metrology[1]. The DPPT-TT:SHE films are transferred onto pre-strained PDMS substrate. After releasing, the buckles of DPPT-TT:SHE film can be obtained. The elastic modulus of film is determined by equation (S2) and the optical images of buckles are shown Supplementary Fig. 5.

$$\frac{\mathrm{E_f}}{(1-v_f^2)} = \frac{3E_s}{(1-v_s^2)}\left(\frac{d}{2\pi h_f}\right)^3 \tag{S2}$$

Where E_f is the Young's modulus of film, v_f is the Poisson's ratio of the film, $\mathrm{E_s}$ is elastic modulus of substrate, v_s is Poisson's ratio of substrate, d is wavelength of buckle and h_f is thickness of the film. In this determination, the elastic modulus of PDMS (E_s) is 2 MPa, Poisson ratio of PDMS (v_s) is 0.5, Poisson ratio of the films (v_f) is 0.35. The thickness of the films is obtained by ellipsometer.

Supplementary Note 3. Calculation method for dielectric constant (*k*)

The dielectric constant (Relative permittivity) is calculated following equation (S3).

$$k = \frac{C_i}{\varepsilon_0} d \qquad \text{(S3)}$$

Where k is dielectric constant, C_i is capacitance per unit area at 1 kHz, ε_0 is permittivity of vacuum and d is thickness of dielectric film. The structure and method for MIM structure are shown at Supplementary Fig. 39.

Supplementary Note 4. Calculation method for surface energies of the neat DPPT-TT, 3:7 blend film, and neat SHE elastomer

The surface free energies are calculated using the Owens-Wendt method[2]:

$$\gamma_c = \gamma_c^p + \gamma_c^d \tag{S4}$$

$$(1 + \cos\theta_l)\gamma_l = 2\left(\sqrt{\gamma_l^d \gamma_c^d} + \sqrt{\gamma_l^p \gamma_c^p}\right) \tag{S5}$$

where γ_c, γ_c^p, and γ_c^d are the total surface energy, polar component and dispersive component of surface energy of testing materials, respectively. Where θ_l, γ_l, γ_l^p, and γ_l^d are the contact angle, total surface energy, polar and dispersive component of surface energy of the test liquid, which are water and diiodomethane. $\gamma_{diiodomethane}$ = 50.8 mJ/m^2, γ_{water} = 72.8 mJ/m^2, γ_{water}^d = 21.8 mJ/m^2 and γ_{water}^p = 51 mJ/m^2.

Supplementary Figures

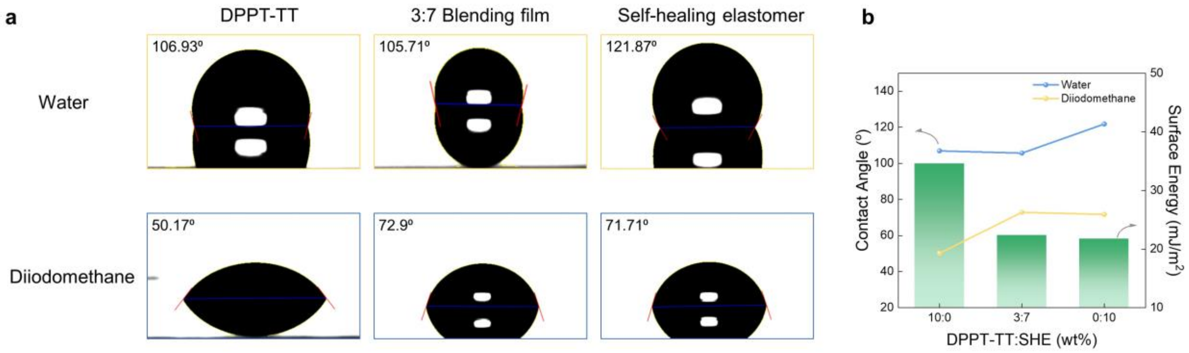

Supplementary Fig. 1. a, Optical images of the contact angles and **b,** surface energies of the neat DPPT-TT, 3:7 blending film and neat self-healing elastomer. The surface energies of neat DPPT-TT, DPPT-TT:SHE (3:7 weight ratio), and neat SHE are 34.55 mJ/m^2, 22.32 mJ/m^2, 21.7 mJ/m^2, respectively.

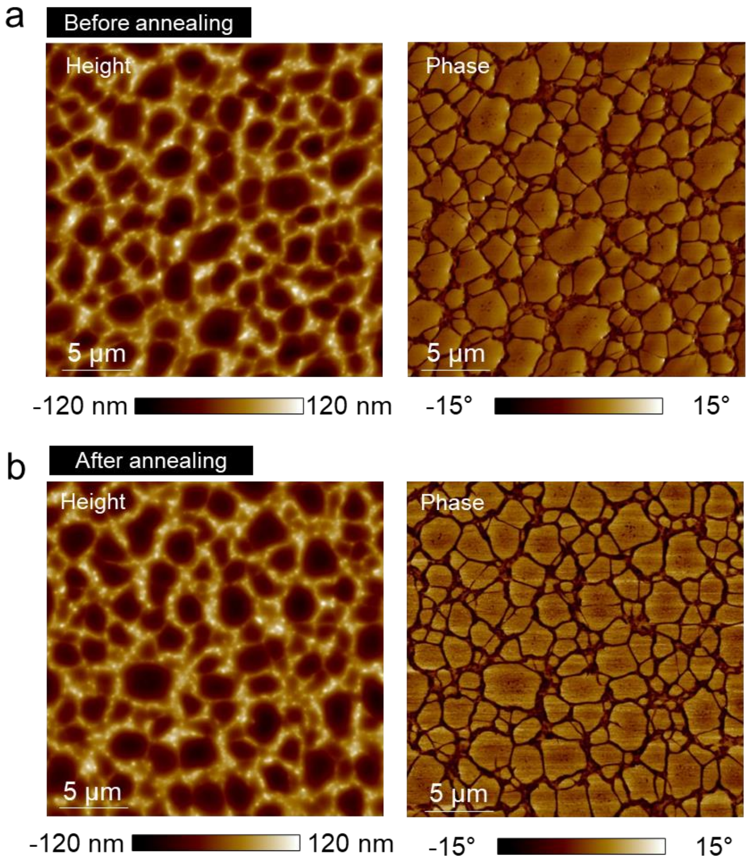

Supplementary Fig. 2. AFM images (left: height, right: phase) of the blend film (3:7 weight ratio, DPPT-TT:SHE) **a,** before and **b,** before (top) and after (bottom) thermal annealing at 80 °C for 30 min.

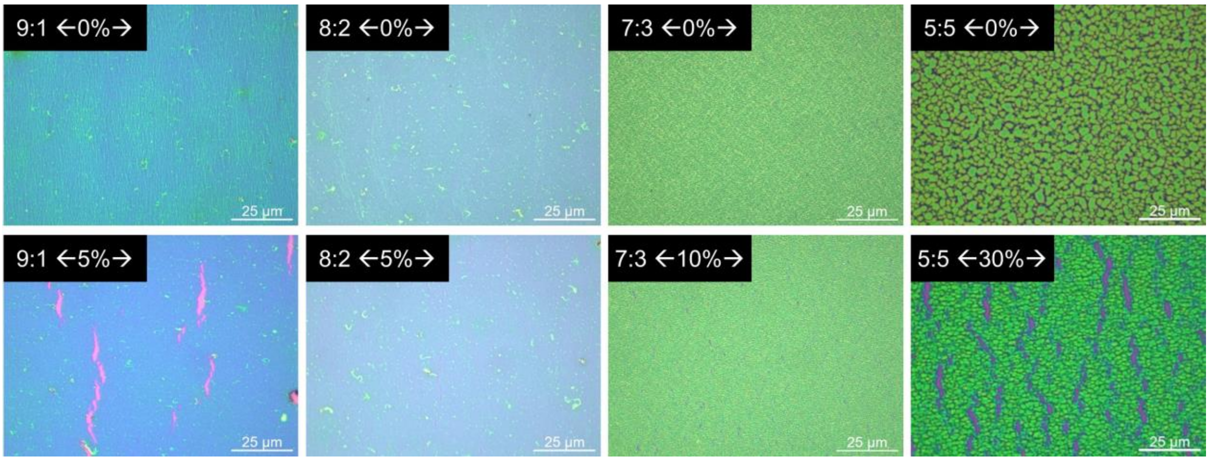

Supplementary Fig. 3. OM images at 0% (top) and crack on-set (bottom) strain of blend films (DPPT-TT:SHE) from 9:1 to 5:5 weight ratio.

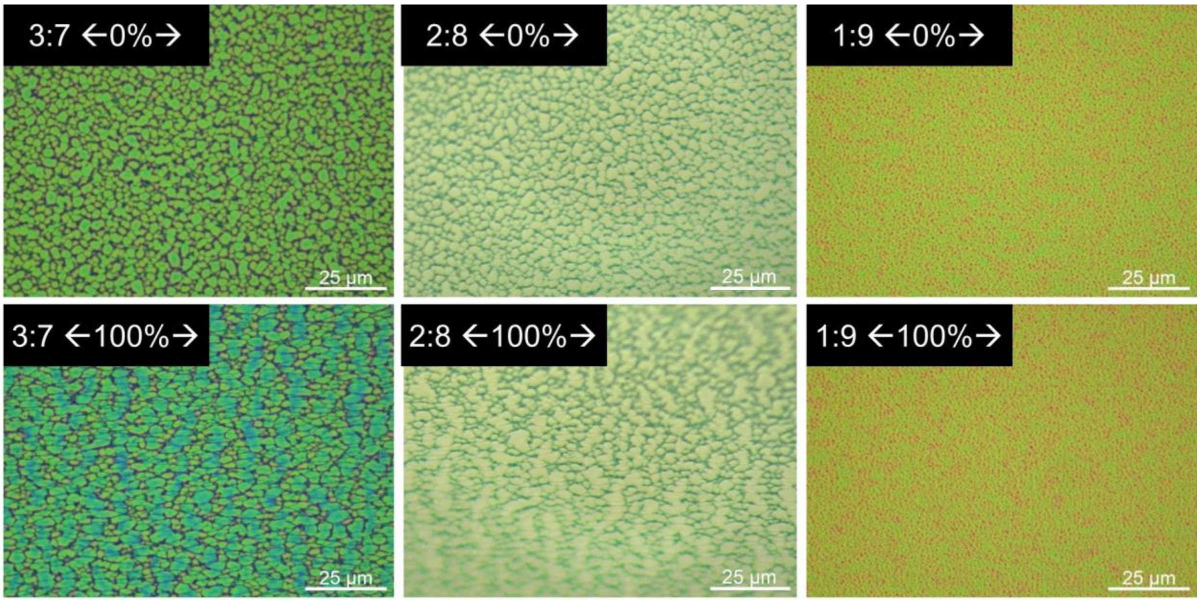

Supplementary Fig. 4. OM images at 0% (top) and 100% (bottom) strain of blend films (DPPT-TT:SHE) with 3:7, 2:8, and 1:9 ratio.

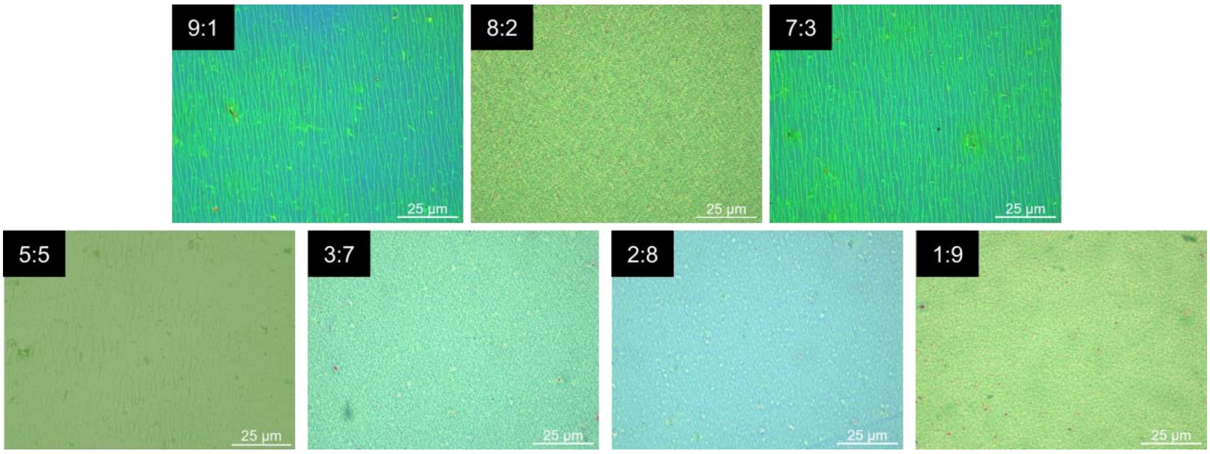

Supplementary Fig. 5. OM images of buckled blend films (DPPT-TT:SHE) with various blending ratios for elastic modulus. The elastic modulus of the films is calculated with equation S2 and Supplementary Note 2. The thickness of all blending films is approximately 100 nm.

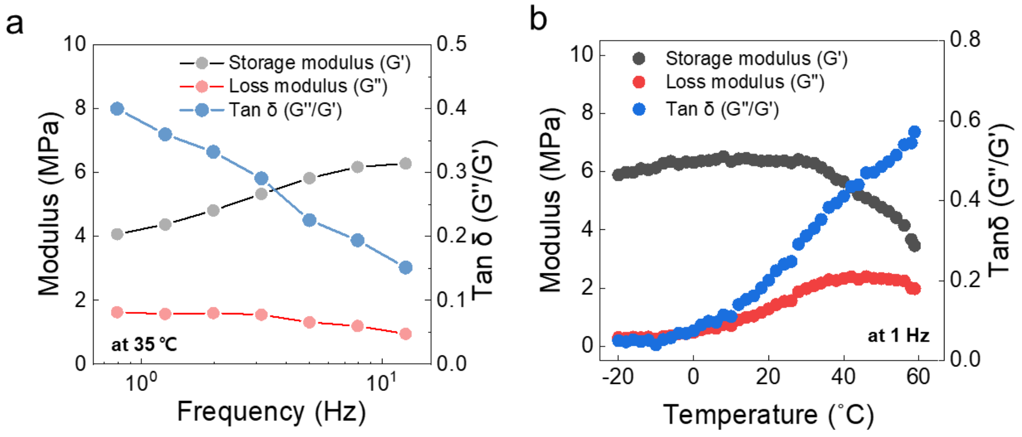

Supplementary Fig. 6. Dynamic mechanical properties of the blend film (3:7, DPPT-TT:SHE). Storage modulus (G'), loss modulus (G'') and tan delta (G''/G') according to **a,** frequency and **b,** temperature, respectively.

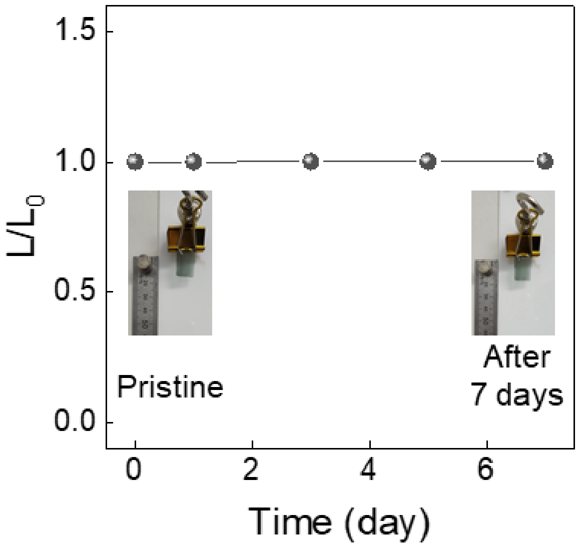

Supplementary Fig. 7. Photographs and length change of DPPT-TT:SHE film suspended under the gravity for 7 days at room temperature.

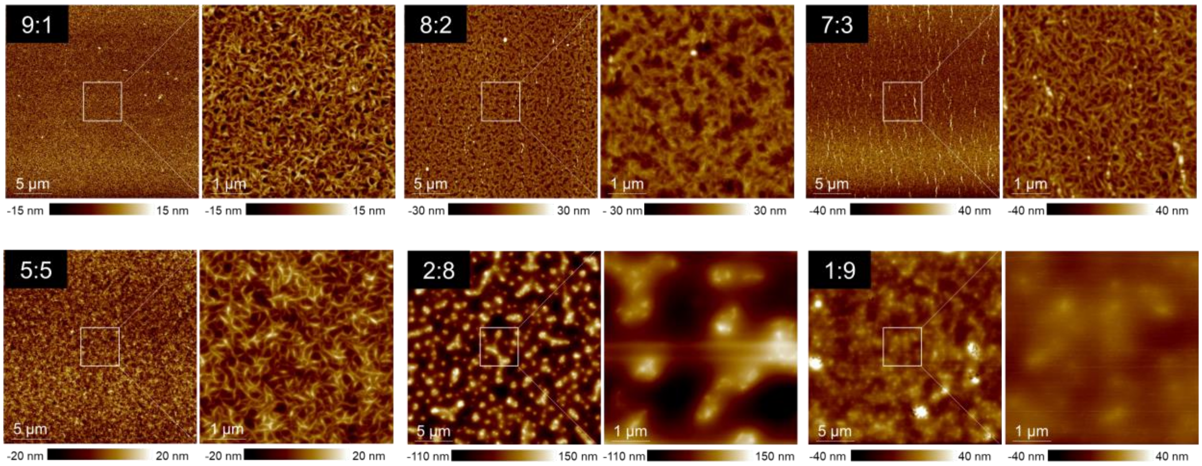

Supplementary Fig. 8. AFM height images of the blend films with various weight ratios (DPPT-TT:SHE, 9:1 to 1:9).

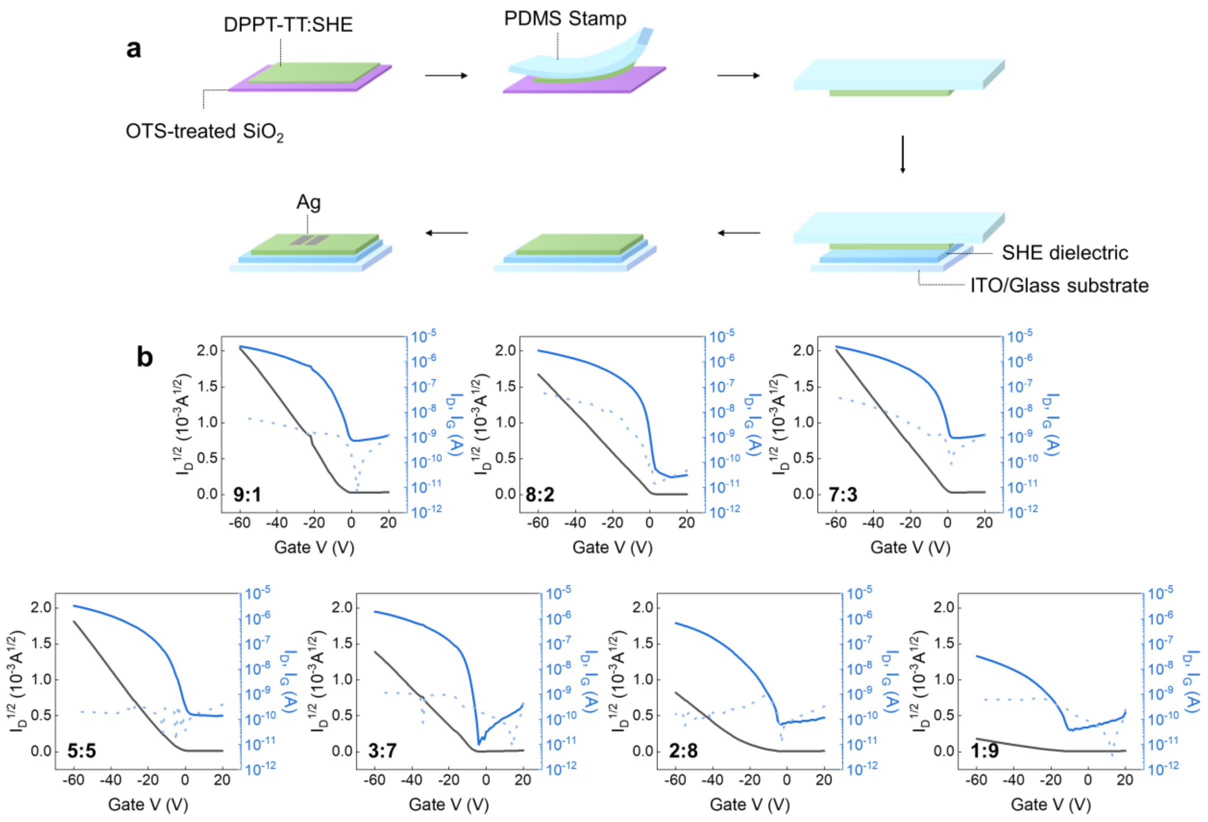

Supplementary Fig. 9. a, Transistor structure that is fabricated on rigid substrate (ITO-glass). **b,** The transfer characteristics of the transistors as a function of blend ratios (DPPT-TT:SHE).

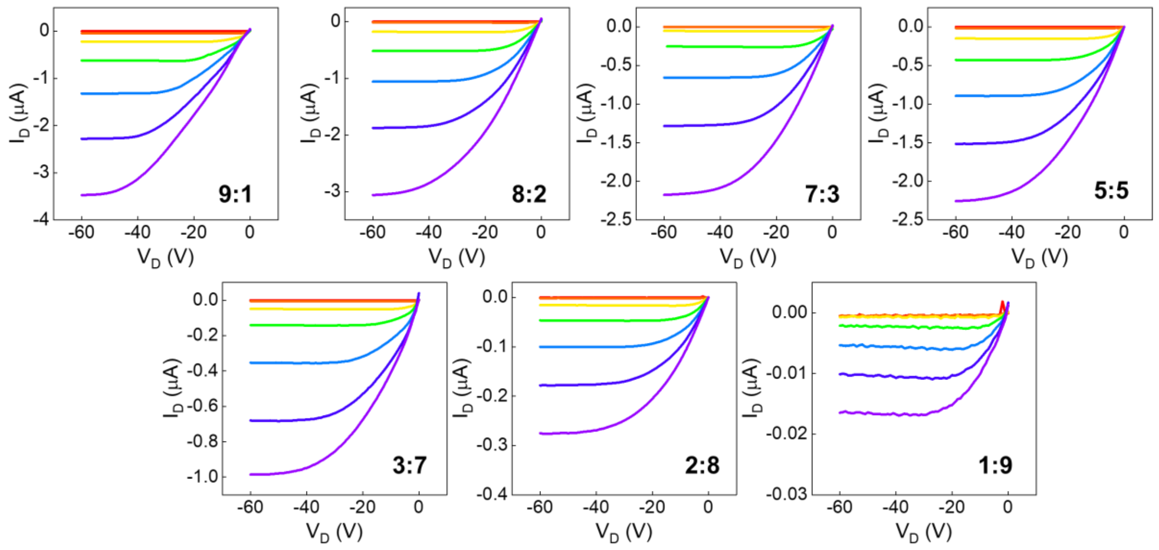

Supplementary Fig. 10. The output characteristics (V_G: 0 to -60 V, step: -10 V) of the blend films with various blend ratios (DPPT-TT:SHE).

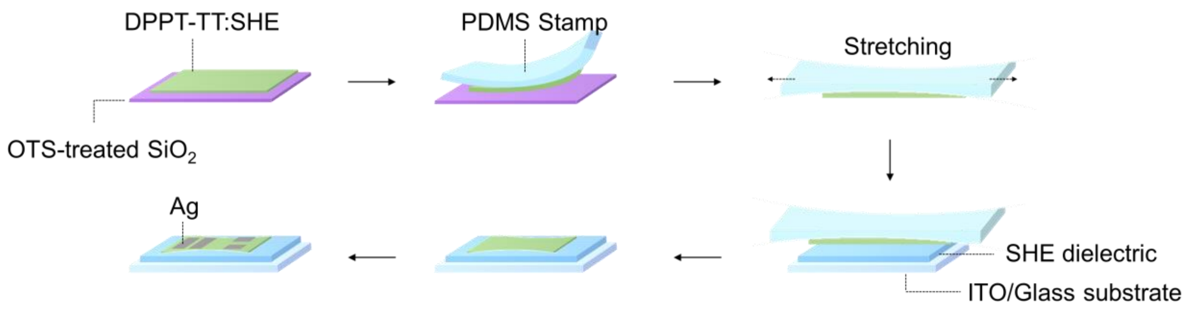

Supplementary Fig. 11. Schematic illustration of transistor structure for electrical measurement. The stretched-semiconducting film was transferred onto SEBS/ITO substrate, and the silver source and drain electrode was then deposited onto the semiconducting films.

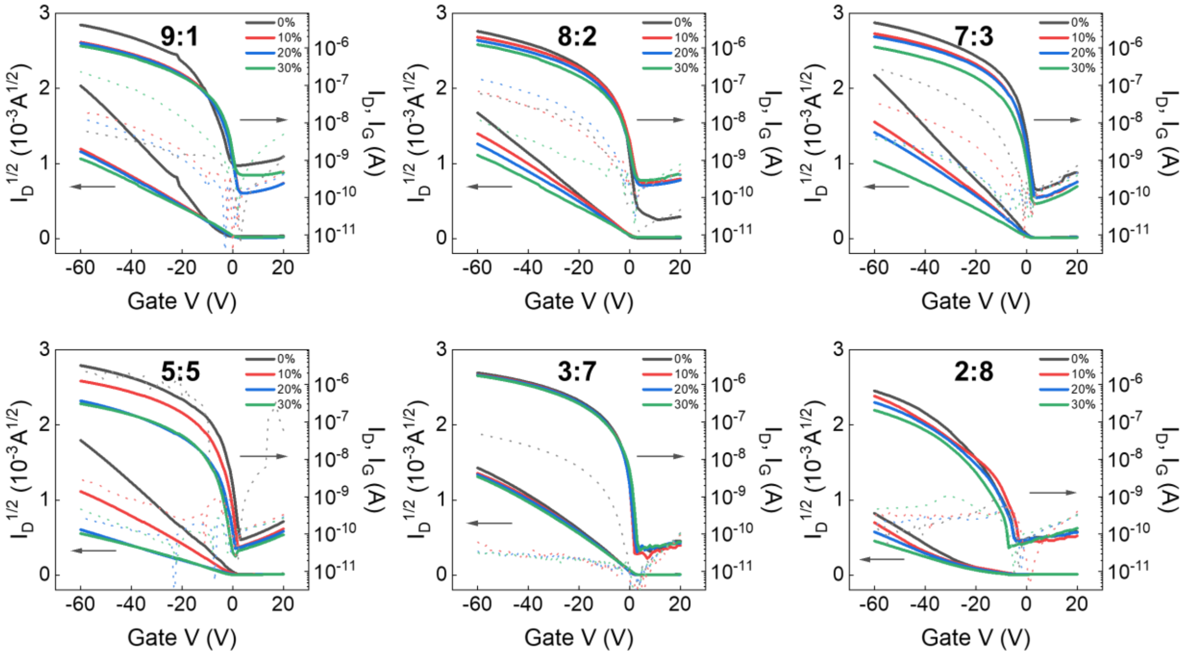

Supplementary Fig. 12. Transfer characteristics of DPPT-TT:SHE films with various blend ratios under 0% to 30% uniaxial strain.

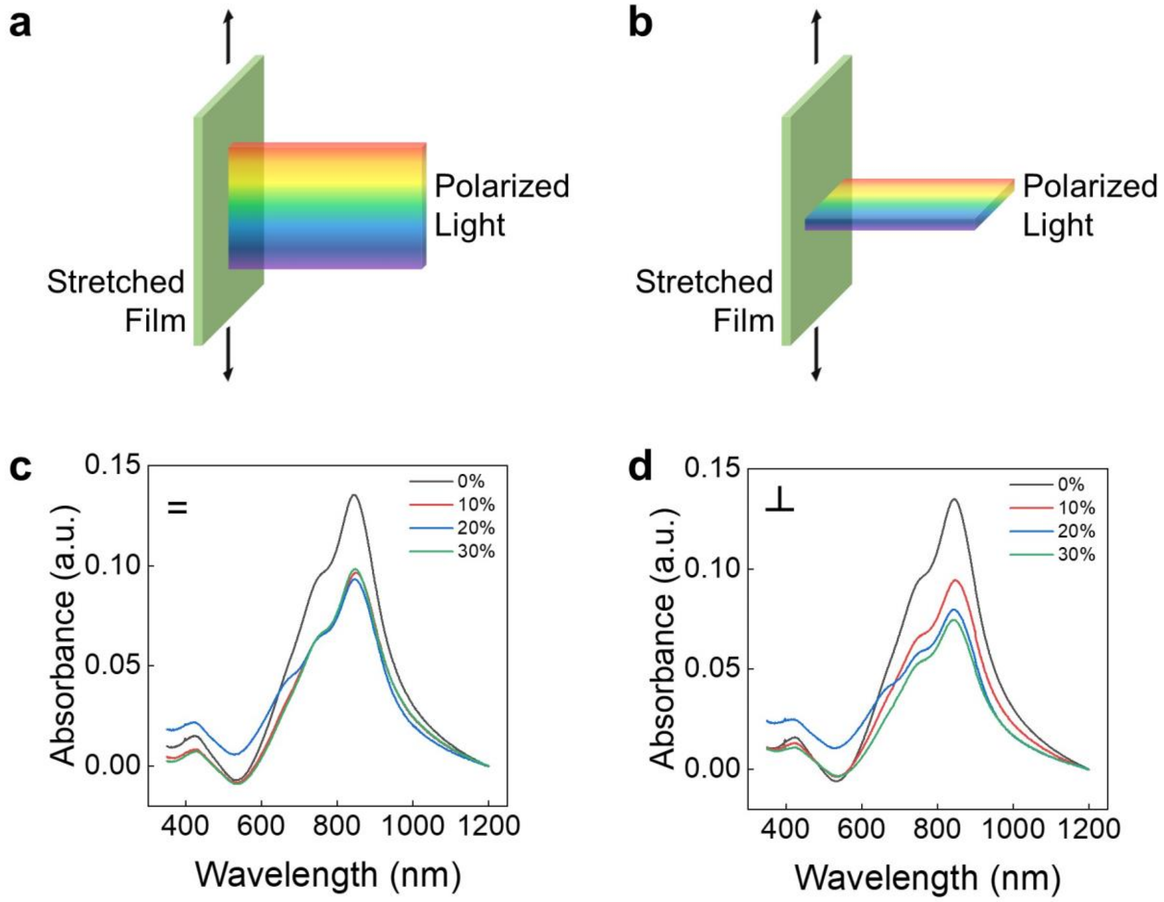

Supplementary Fig. 13. Schematic illustrations of polarized UV-vis-NIR characterization of the films. Polarized light in **a,** parallel and **b,** perpendicular directions to the stretching direction. Polarized UV-vis-NIR spectra of the blend film under 0% to 30% strain in **c,** parallel and **d,** perpendicular directions to the stretching direction.

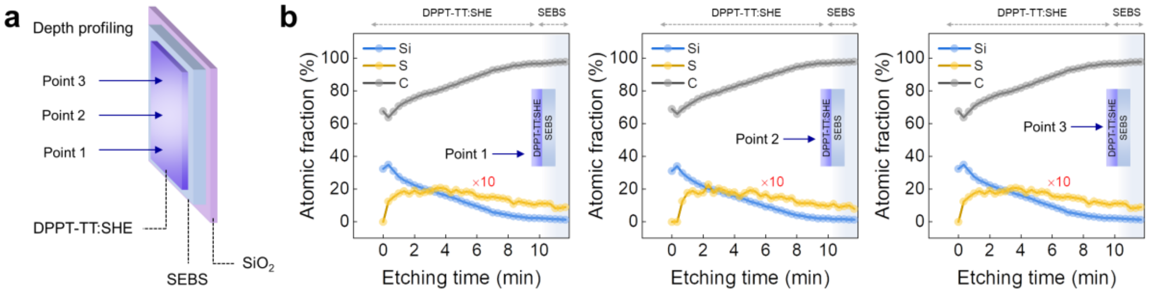

Supplementary Fig. 14. a, Schematic illustration of the sample structure for XPS analysis. The DPPT-TT:SHE blending film, in a 3:7 ratio, was spin-coated onto OTS-treated SiO_2. This blending film was then transferred onto an SEBS/SiO_2 substrate, where the SEBS layer served as a support for the separation of Si atoms from both DPPT-TT:SHE film and SiO_2 substrate. Notably, the SEBS consists only of C and H atoms. **b,** XPS depth profiling of atomic fraction in DPPT-TT:SHE films with three different point.

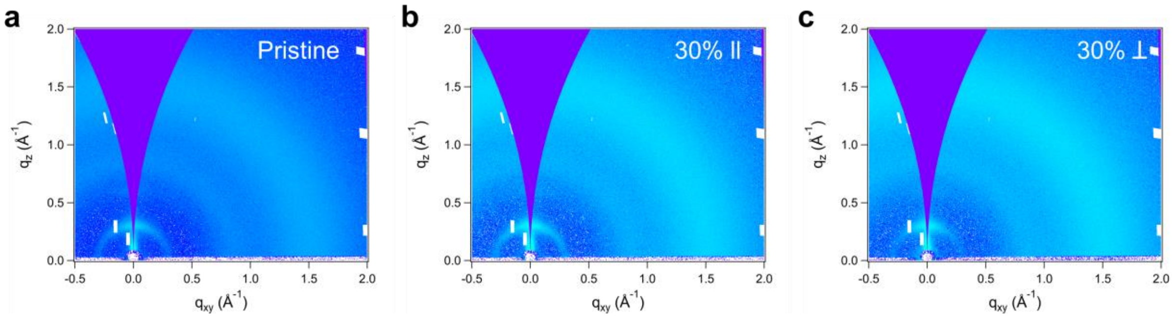

Supplementary Fig. 15. Two-dimensional GIXD patterns of the blend film (DPPT-TT:SHE=3:7 weight ratio) at **a,** pristine and stretched films under 30% strain in **b,** parallel, and **c,** perpendicular to the incident beam orientation.

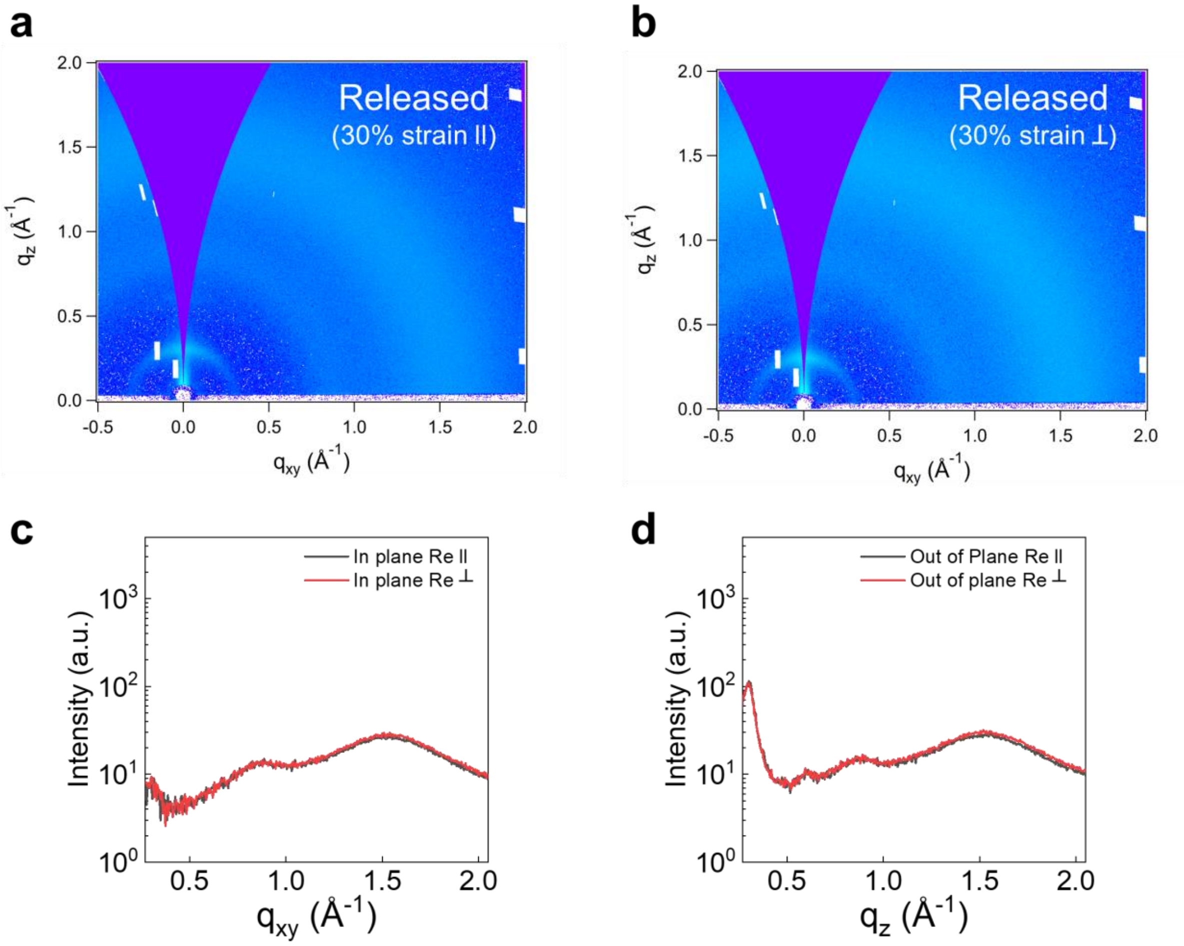

Supplementary Fig. 16. Two-dimensional GIXD patterns of the blend film (DPPT-TT:SHE=3:7 weight ratio) after stretching (30 % strain) in **a,** parallel and **b,** perpendicular to stretching-releasing direction. The intensity line cuts for the releasing film along **c,** in plane and **d,** out of plane of the films.

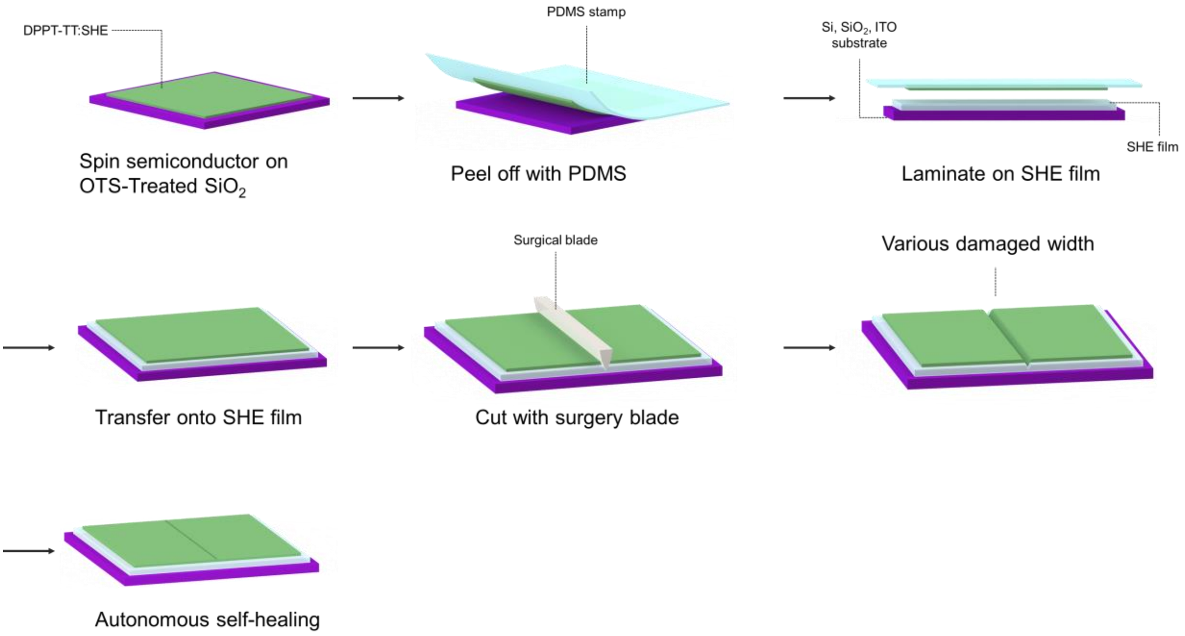

Supplementary Fig. 17. Schematic illustrations of autonomous self-healing process. The semiconducting film is transferred onto SHE film. We used various substrates such as Si, SiO_2, and ITO-glass for device fabrication and materials analysis. The semiconducting film was cut using a surgery blade, resulted in microscale damage width up to 5μm.

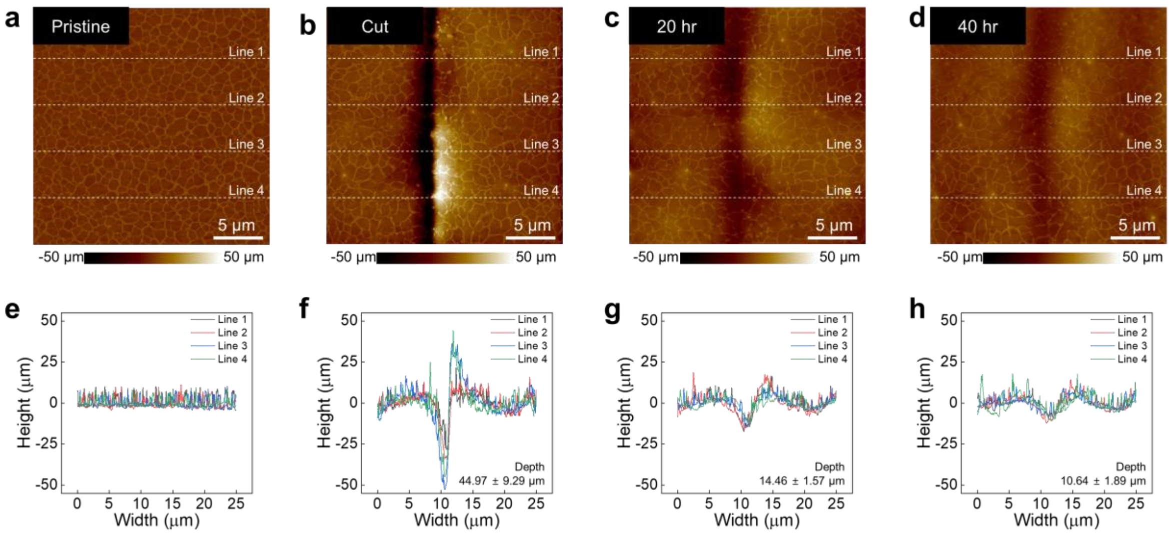

Supplementary Fig. 18. AFM height images of the DPPT-TT:SHE films for self-healing process: **a,** pristine **b,** after cut, **c,** after 20 hours and **d,** after 40 hours states. Line height profiling of the AFM height image **e,** pristine, **f,** after cut, **g,** after 20 h and **h,** after 40 h states. As time flows, the cutting line was healed without any treatment.

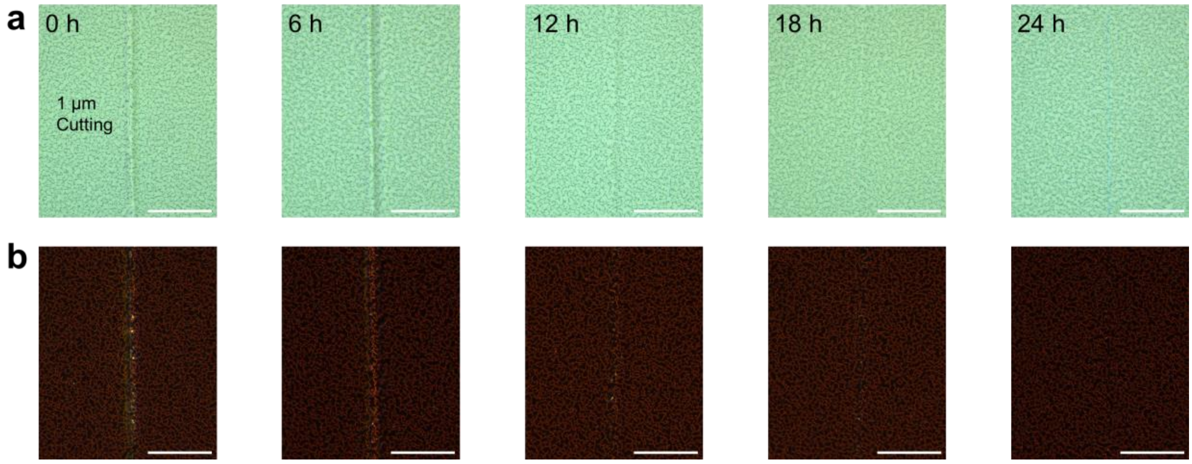

Supplementary Fig. 19. Optical images of about 1 μm damaged area with time flows at room temperature: **a,** bright field images and **b,** dark field images. Scale bar: 50 μm.

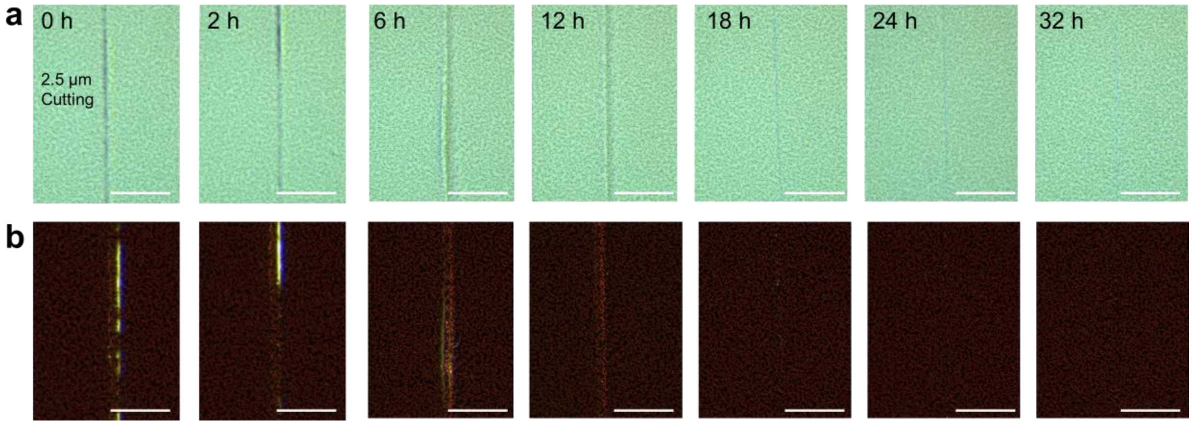

Supplementary Fig. 20. OM images of the damaged blend film (damage width: 2.5 μm) as a function of healing time at room temperature; **a,** bright field images and **b,** dark field images. Scale bar: 50 μm.

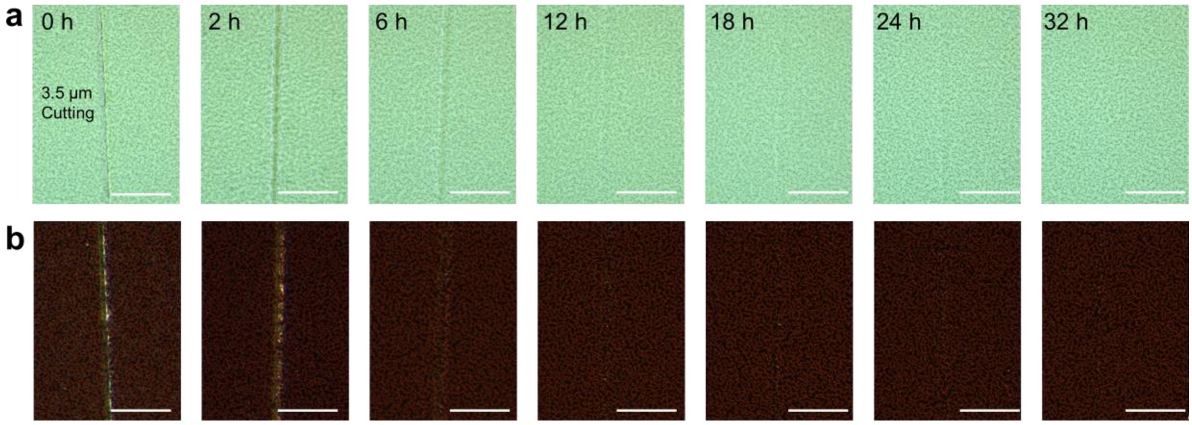

Supplementary Fig. 21. OM images of the damaged blend film (damage width: 3.5 μm) as a function of healing time at room temperature; **a,** bright field images and **b,** dark field images. Scale bar: 50 μm.

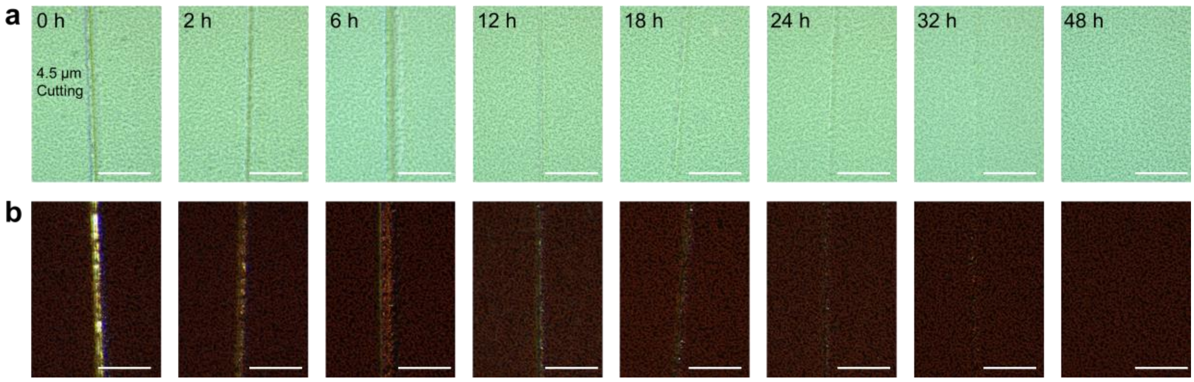

Supplementary Fig. 22. OM images of the damaged blend film (damage width: 4.5 μm) as a function of healing time at room temperature; **a,** bright field images and **b,** dark field images. Scale bar: 50 μm.

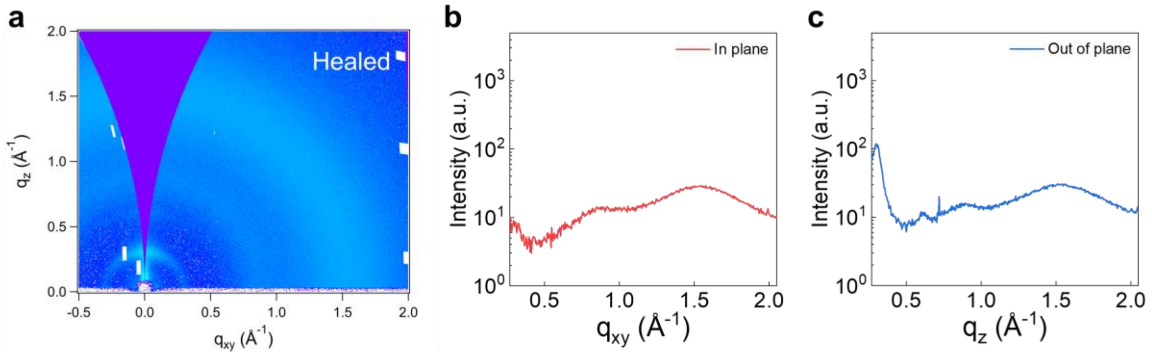

Supplementary Fig. 23. a, Two dimensional GIXD pattern of the healed semiconducting film. **b,** The intensity line cuts along in plane and **c,** out of plane of the healed film.

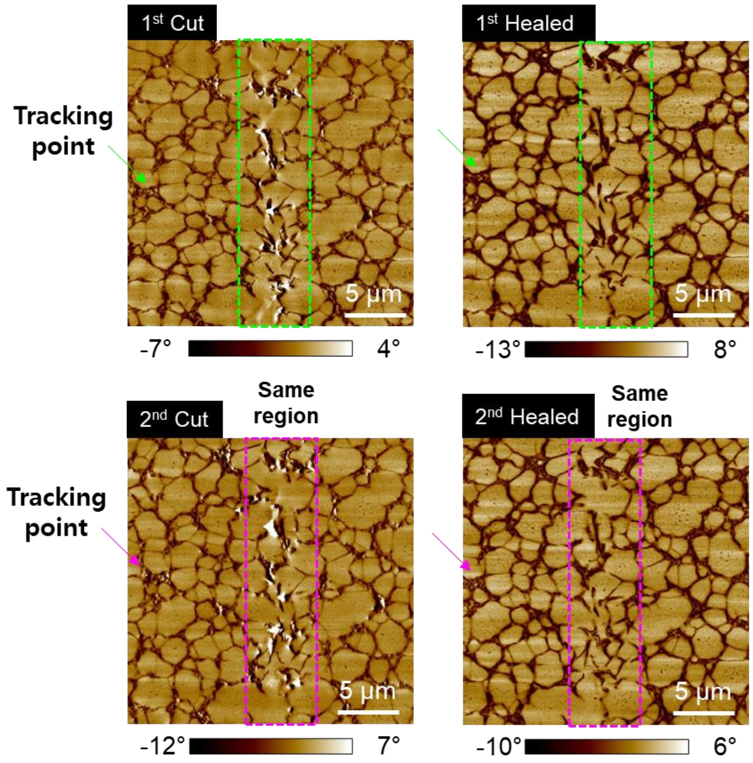

Supplementary Fig. 24. AFM phase images of the blend film (3:7, DPPT-TT:SHE) during multiple cutting and healing at same region.

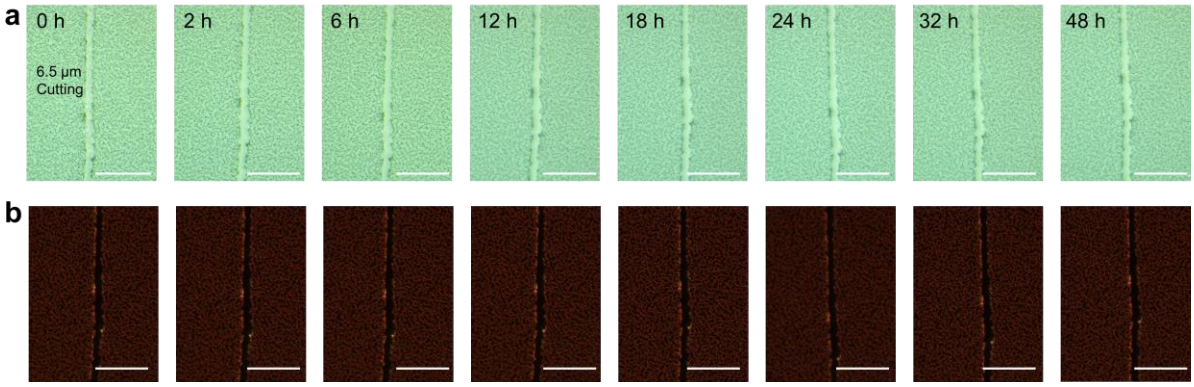

Supplementary Fig. 25. OM images of the damaged blend film (damage width: 6.5 μm) as a function of healing time at room temperature; **a,** bright field images and **b,** dark field images. Scale bar: 50 μm.

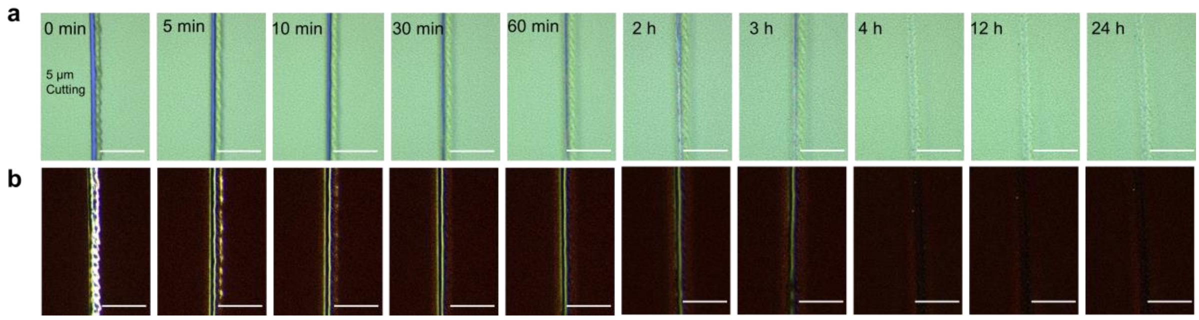

Supplementary Fig. 26. OM images of the damaged blend film (damage width: 5 μm) as a function of the time of heat treatment at 40 ºC: **a,** bright field images and **b,** dark field images. Scale bar: 50 μm.

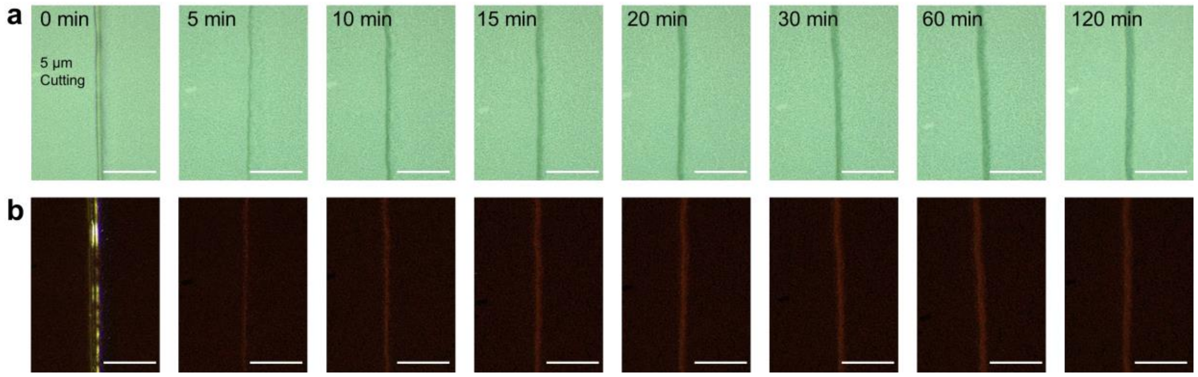

Supplementary Fig. 27. Optical images of about 5 μm damaged area with time flows during Chloroform solvent vapor treatment at room temperature after cutting: **a,** bright field images **b,** dark field images. Scale bar: 50 μm.

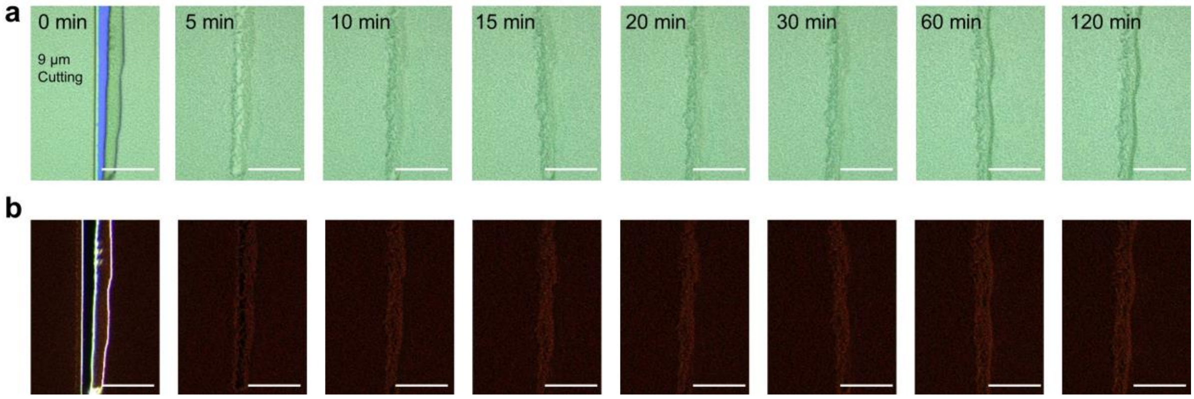

Supplementary Fig. 28. Optical images of about 9 µm damaged area with time flows during Chloroform solvent vapor treatment at room temperature after cutting: **a,** bright field images **b,** dark field images. Scale bar: 50 µm.

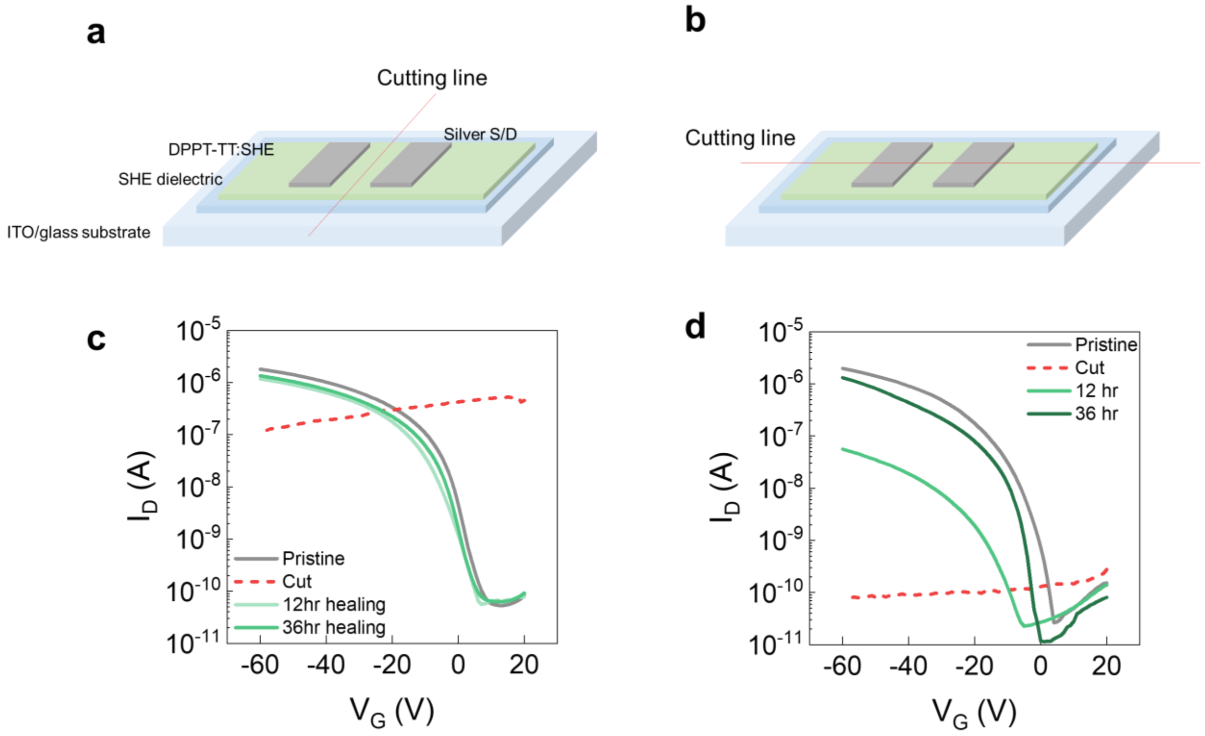

Supplementary Fig. 29. Schematic illustration of cutting process. **a,** Perpendicular and **b,** parallel cutting direction to channel direction. The transfer characteristics (V_D: -60 V) of **c,** perpendicular and **d,** parallel cutting direction to the channel direction with 1 μm cutting width.

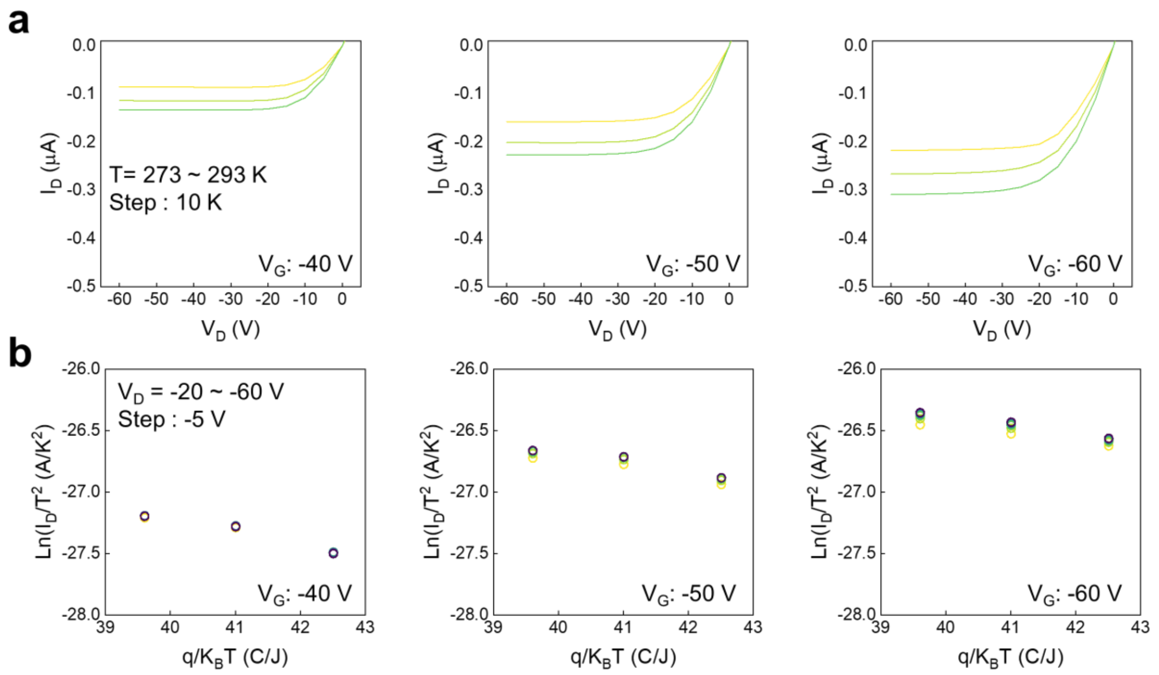

Supplementary Fig. 30. a, Output characteristics as a function of temperature with different gate voltage. **b,** Arrhenius fitting the $Ln(I_D/T^2)$ versus q/K_BT, where q and K_B are electrical constant and Boltzmann constant, respectively. Calculated effective Schottky barrier height against the drain voltage using below equation: $I_D = AA^*T^2 \exp(-q\Phi_B/k_BT)$, where A and A^* are barrier area and Richardson constant, respectively.

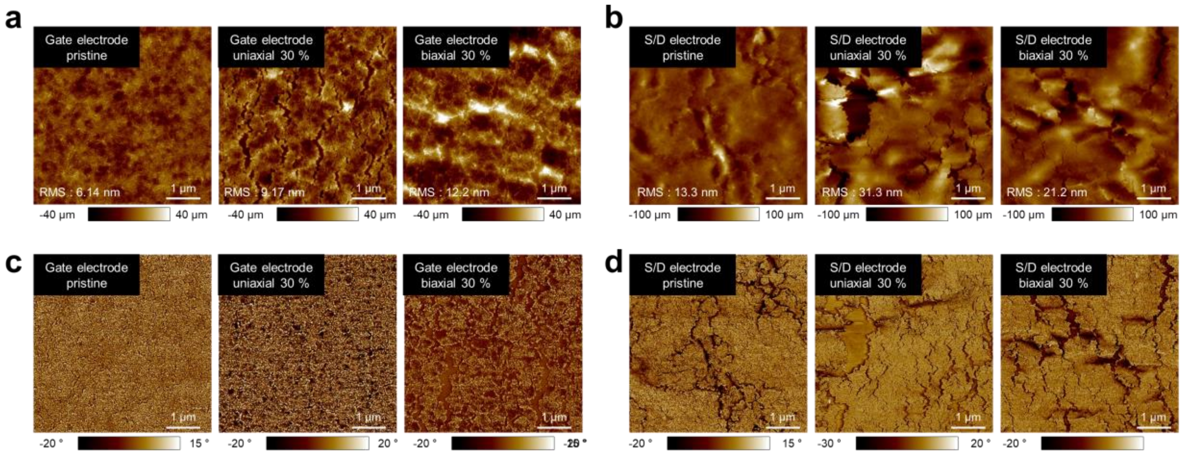

Supplementary Fig. 31. AFM **a,b,** height and **c,d,** phase images of Ag metallized SHE substrate for gate and DPPT-TT:SEBS semiconductor for source and drain on 0% (left), 30% uniaxial (middle), and biaxial (right) strains, respectively.

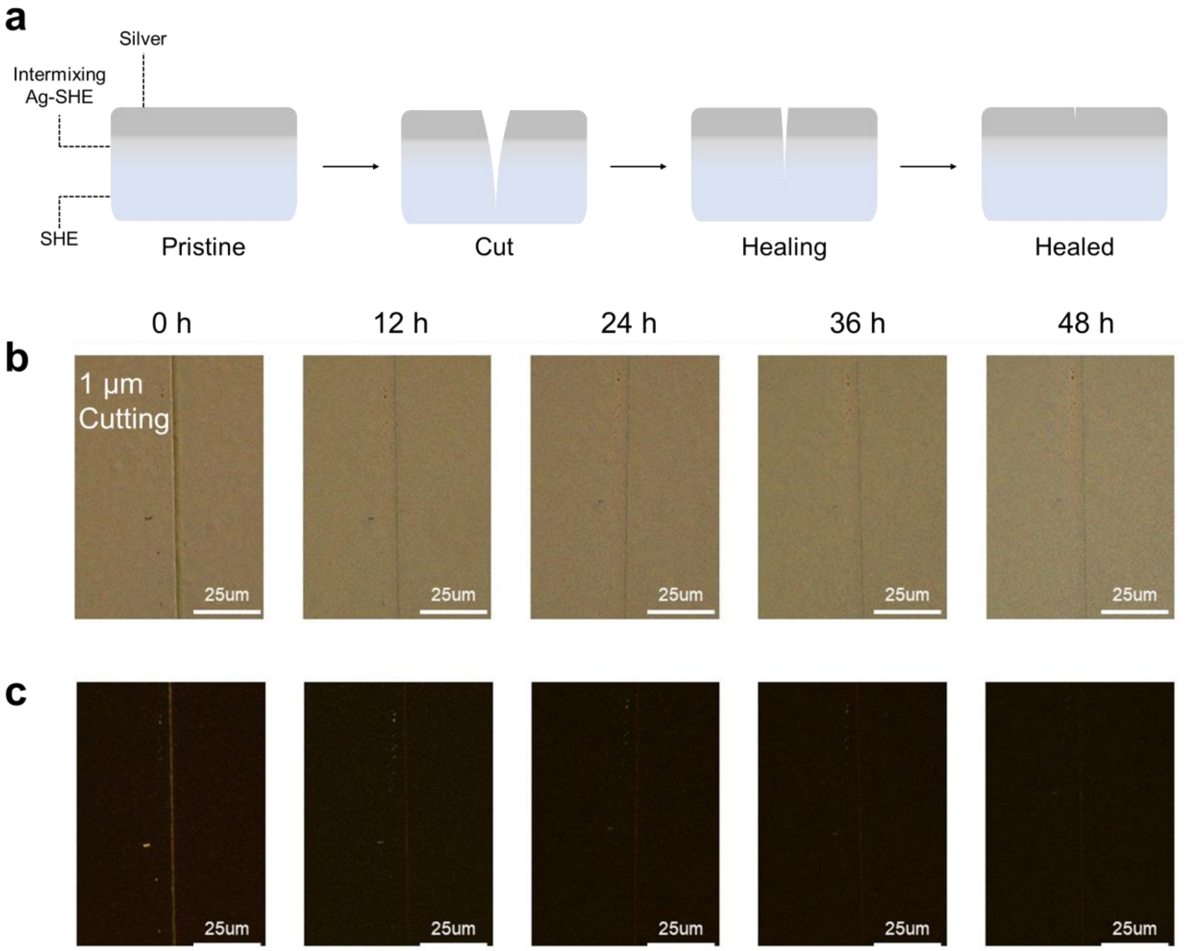

Supplementary Fig. 32. a, Schematic illustration of silver electrode healing on SHE substrate. The deposited-silver on SHE substrate was cut by surgical blade. After cutting, the silver was observed using optical microscope without any treatment. Optical images of silver electrode healing on SHE substrate at room temperature with time flows: **b,** Bright field images and **c,** dark field images. Cutting width is about 1 μm.

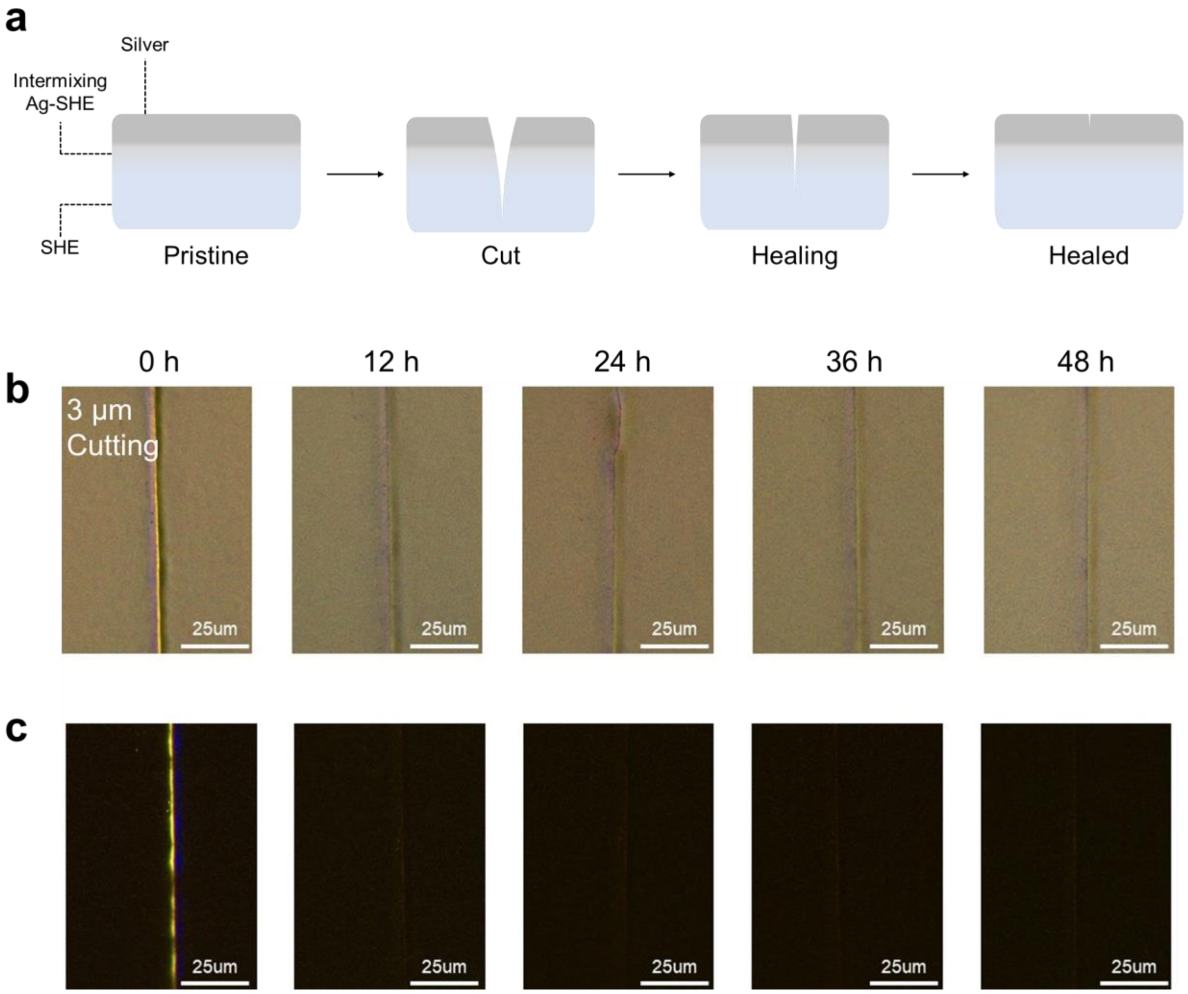

Supplementary Fig. 33. a, Schematic illustration of silver electrode healing on SHE substrate. Optical images of silver electrode healing on SHE substrate at room temperature with time flows: **b,** bright field images and **c,** dark field images. Cutting width is about 3 μm.

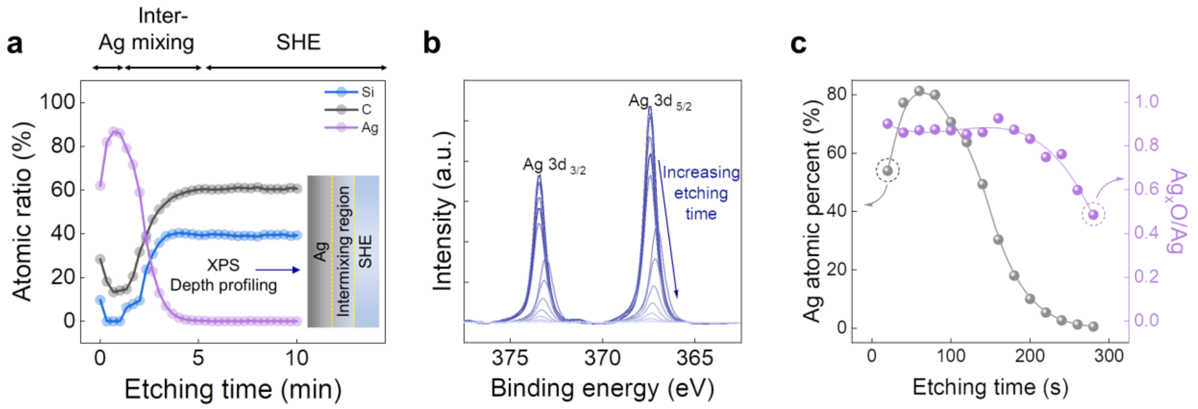

Supplementary Fig. 34. a, XPS depth profiling of Ag/SHE film. **b,** XPS spectra of Ag 3d peak and **c,** Ag atomic percent and Ag_xO/Ag ratio as a function of etching time.

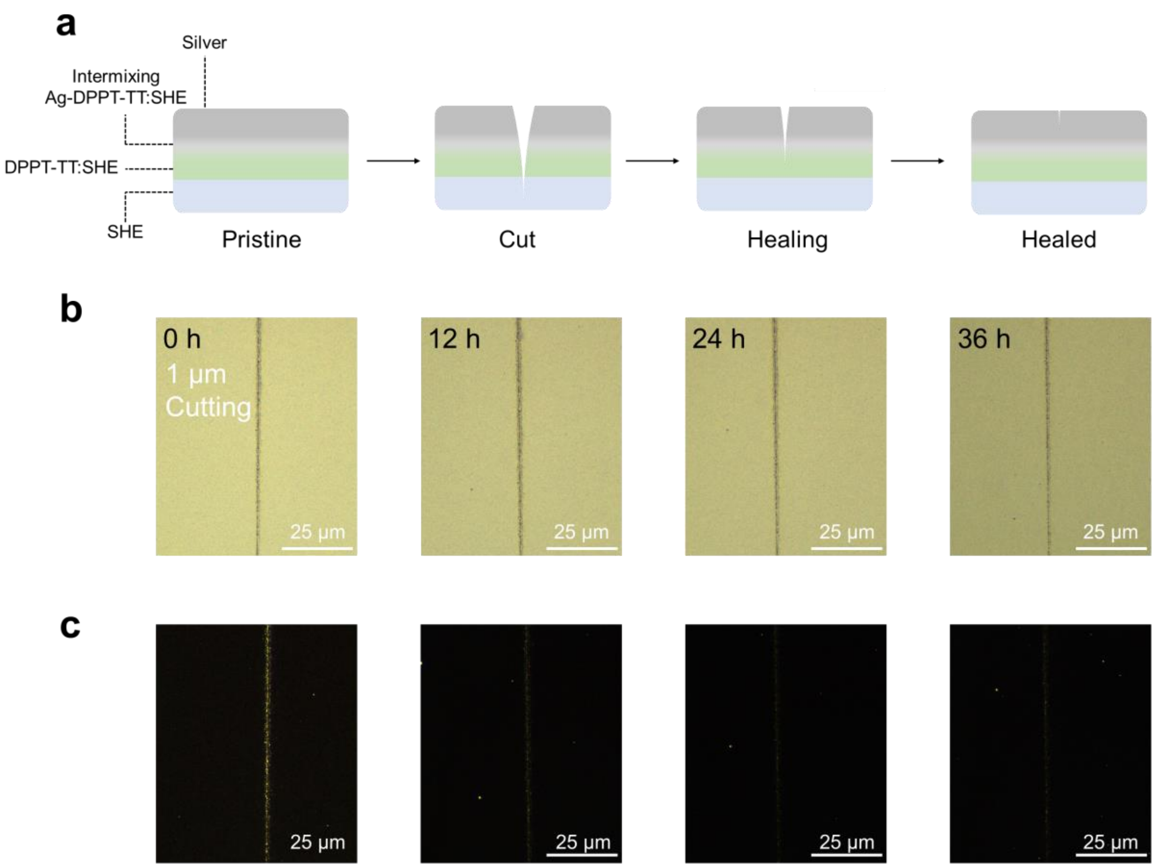

Supplementary Fig. 35. a, Schematic illustration of cut and autonomous-healing process of silver on DPPT-TT:SHE semiconducting film for source/drain electrode. The deposited silver was cut using surgical blade. Optical images of silver electrode healing on semiconductor at room temperature with time flows: **b,** bright field images and **c,** dark field images. Cutting width is about 1 μm.

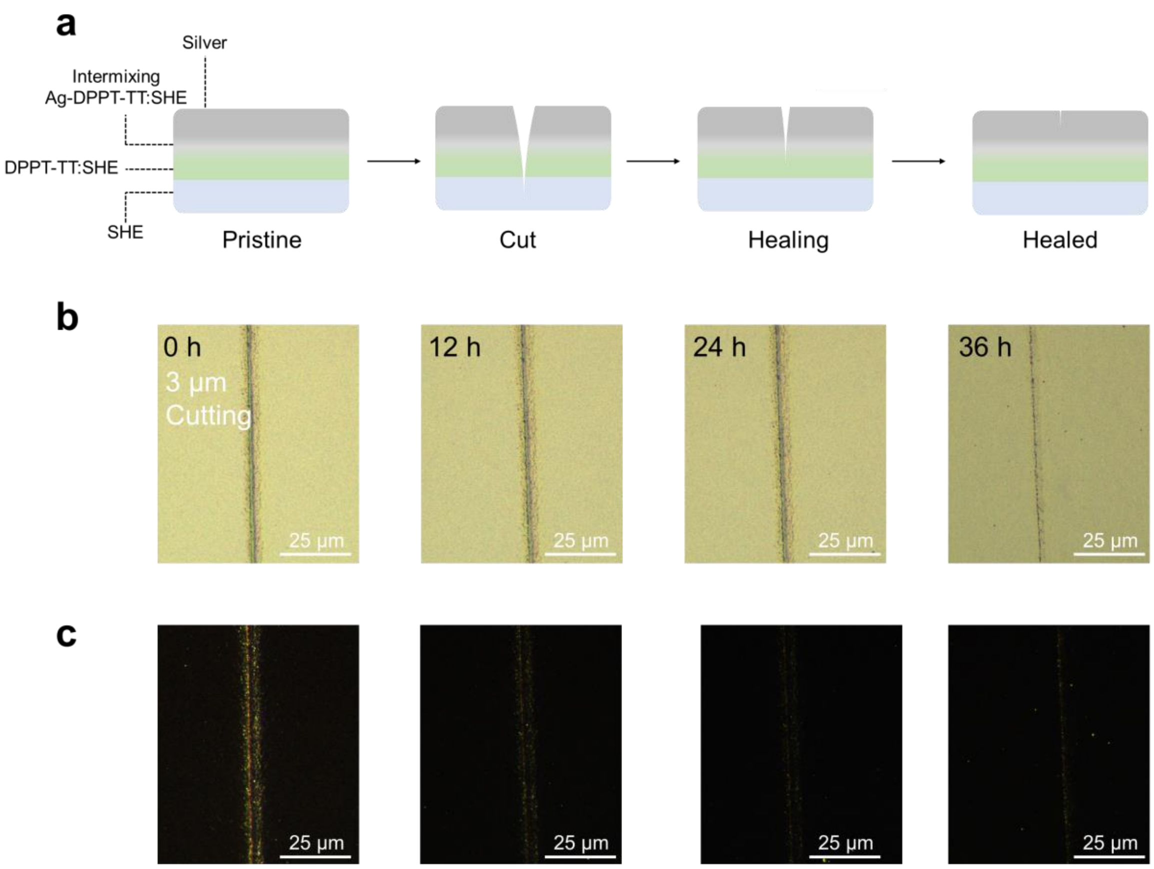

Supplementary Fig. 36. a, Schematic illustration of cut and autonomous-healing process of silver on DPPT-TT:SHE semiconducting film for source/drain electrode. The deposited silver was cut using surgical blade. Optical images of silver electrode healing on semiconductor at room temperature with time flows: **b,** bright field images and **c,** dark field images. Cutting width is about 3 μm.

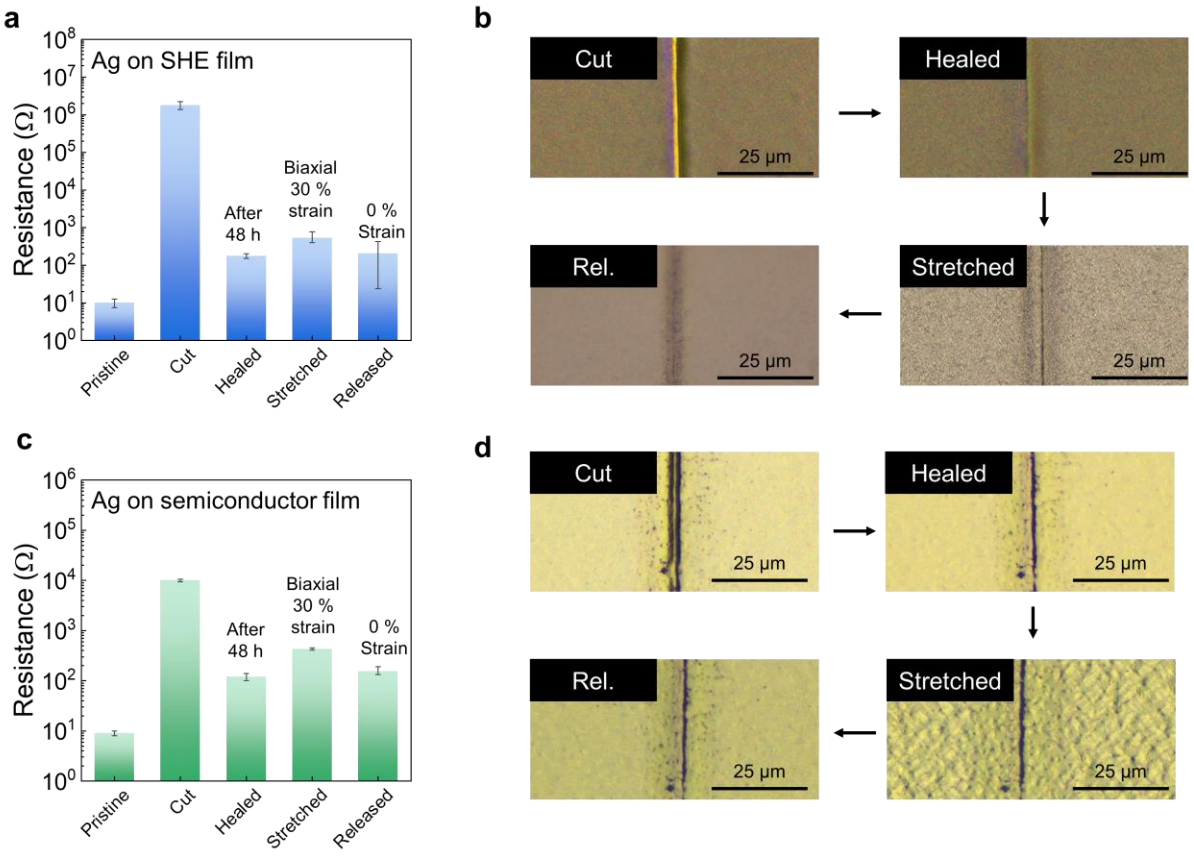

Supplementary Fig. 37. a, Resistance changes (n = 3) and **b,** OM images of silver electrode on SHE films for a healing cycle in sequence of cutting (3 μm cut width), healing (after 48 h), stretching (30 % biaxially strain after healing) and releasing strain (0% strain). **c,** Resistance changes (n = 3) and **d,** OM images of silver electrode for a healing cycle in sequence of cutting (3 μm cut width), healing (after 48 h), stretching (30 % biaxially strain after healing) and releasing strain (0% strain). All processes were conducted at room temperature.

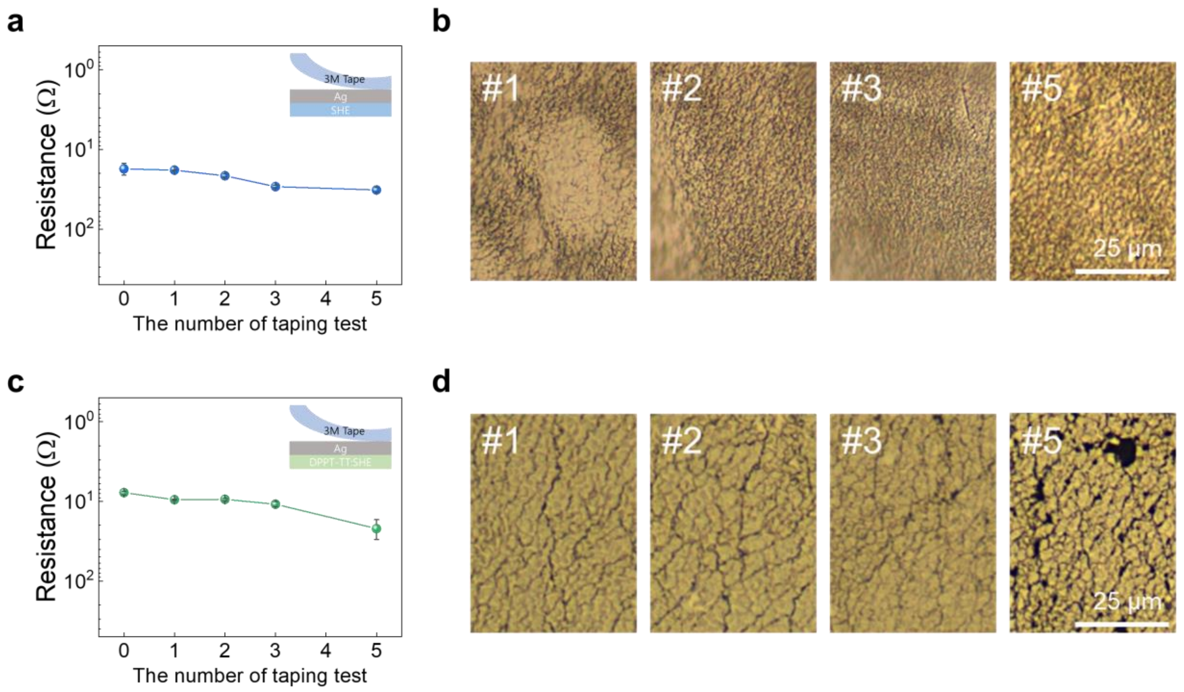

Supplementary Fig. 38. Resistance change of silver electrode (80 nm thick) on **a,** SHE and **c,** DPPT-TT:SHE film as a function of the number of taping test. The inset illustration shows Ag electrode delamination test using 3M^{TM} tape. OM images of silver electrode on **b,** SHE and **d,** DPPT-TT:SHE film with the number of taping test. The electrical conductivity is still remained, even morphology is changed.

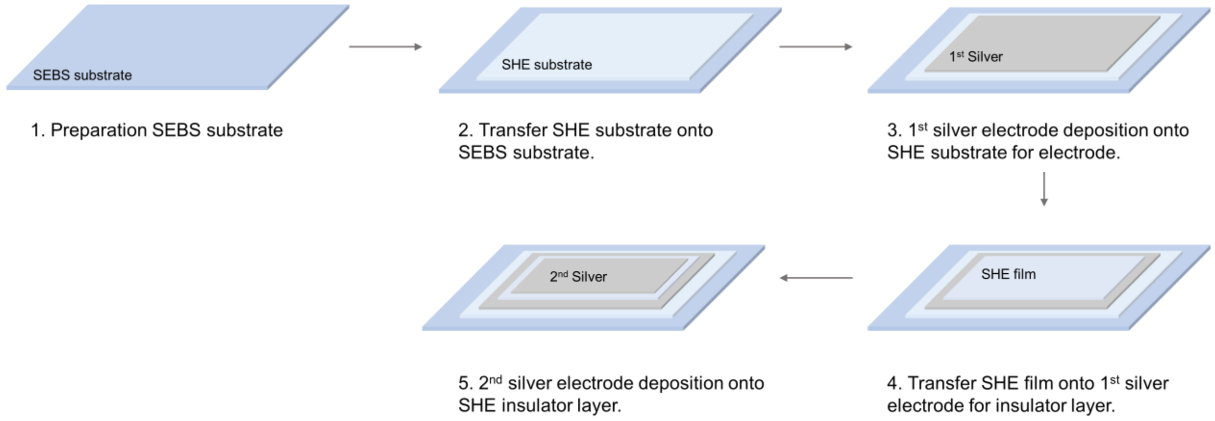

Supplementary Fig. 39. Fabrication steps for metal-insulator-metal (MIM) structure. The SHE substrate (1.0 μm) on OTS-treated SiO_2/Si wafer was directly transferred onto SEBS substrate. After transfer, the 1st silver (80 nm) was deposited on to SHE substrate. The another SHE film (1.5 μm) on OTS-treated SiO_2/Si wafer was directly transferred onto 1st silver electrode. The 2nd silver electrode (80 nm) was deposited onto SHE films.

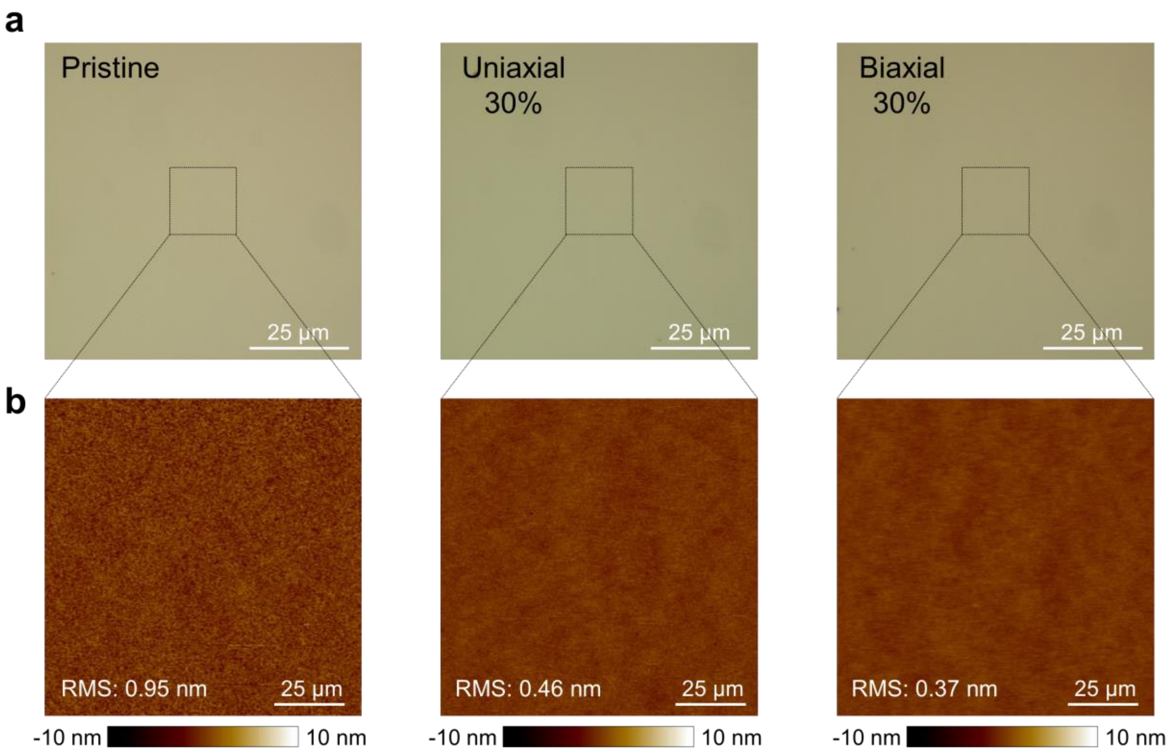

Supplementary Fig. 40. a, OM and **b,** AFM height images of SHE dielectric films on 0%, 30% uniaxial and 30% biaxially strain.

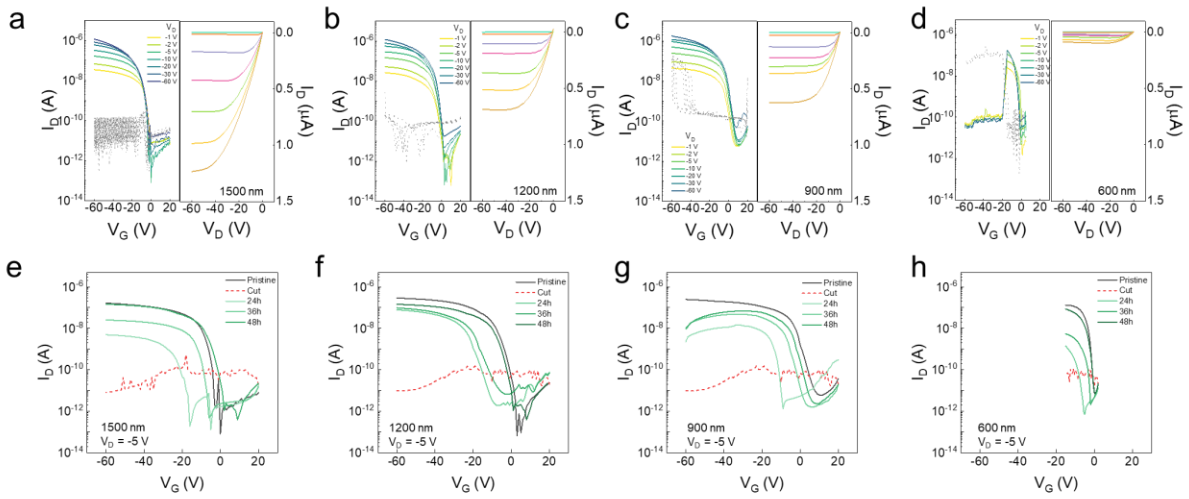

Supplementary Fig. 41. a-d, Transfer and output curves of self-healing transistors with various dielectric thicknesses and different drain voltage. **e-h,** Transfer curves of the self-healing transistors operating at -5 V_D in -60 to 20 V_G while self-healing process for 48 h.

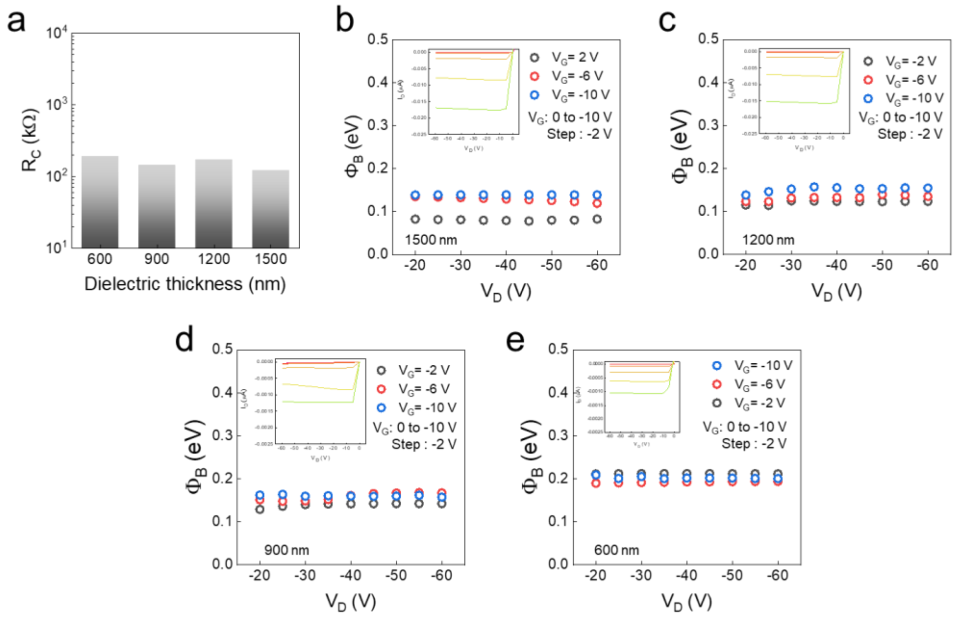

Supplementary Fig. 42. a, Contact resistances and **b-e,** current injection barriers of the self-healing transistor as a function of dielectric thickness ranging from 1,500 nm to 600 nm. Inset of b-e are the output characteristics in low voltage operation.

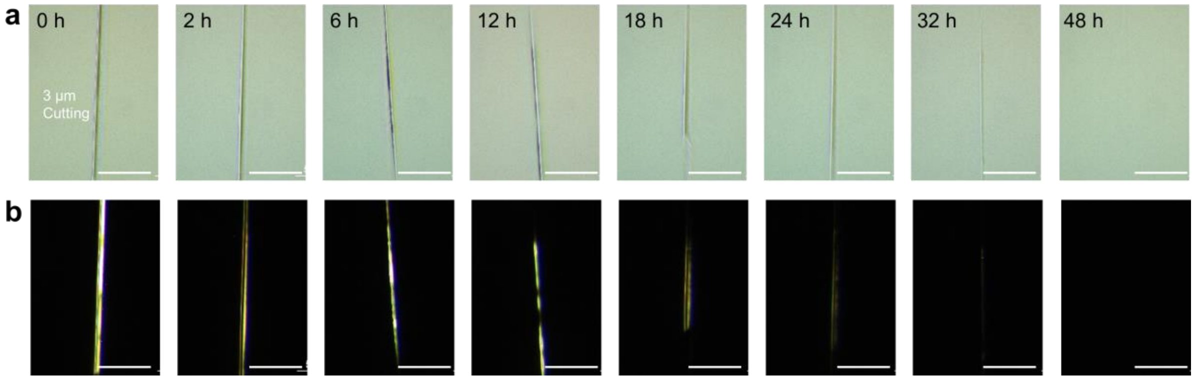

Supplementary Fig. 43. OM images of SHE dielectric film for autonomous healing as a function of time at room temperature: **a,** bright field images and **b,** dark field images. Cutting width is about 3 μm.

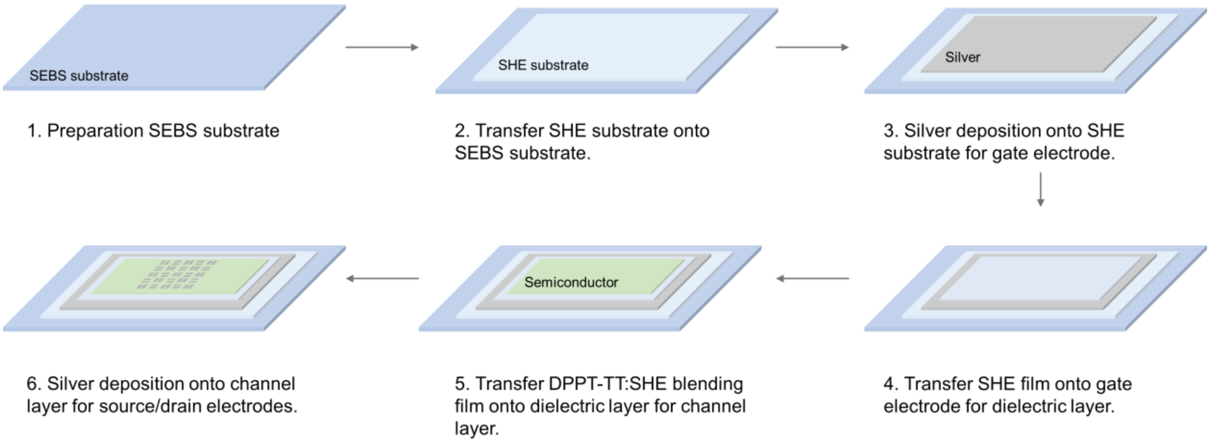

Supplementary Fig. 44. Fabrication steps for stretchable and self-healable passive arrays. The silver gate (80 nm) was deposited onto SHE substrate (1.0 μm) with thermal deposition. After fabrication of gate, SHE dielectric (1.5 μm) and semiconductor (100 nm) on OTS-treated SiO_2/Si wafer was directly transferred onto gate electrode. Finally, silver S/D electrodes (80 nm) were deposited onto semiconductor.

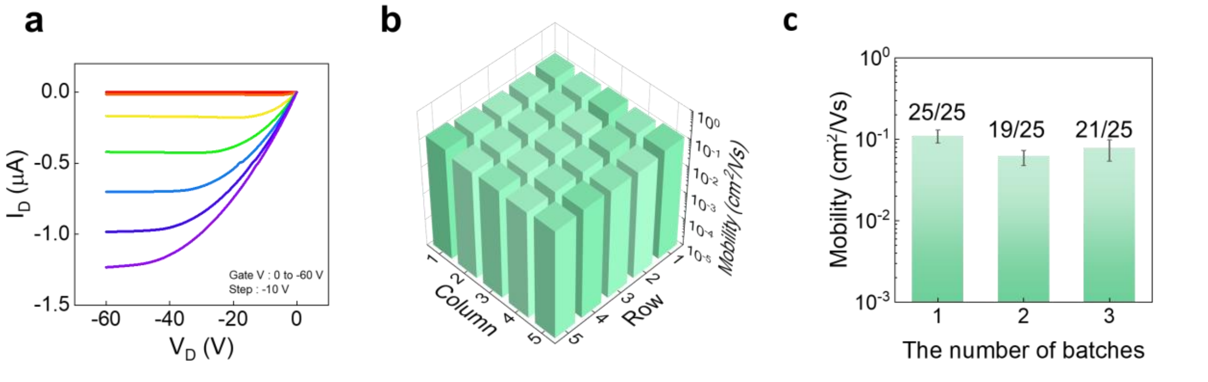

Supplementary Fig. 45. a, Output characteristic and **b,** field effect saturation mobility mapping of fully stretchable and self-healable passive arrays. **c,** Mobility deviation in three different batches. All error bars were calculated using three samples in each of three batches.

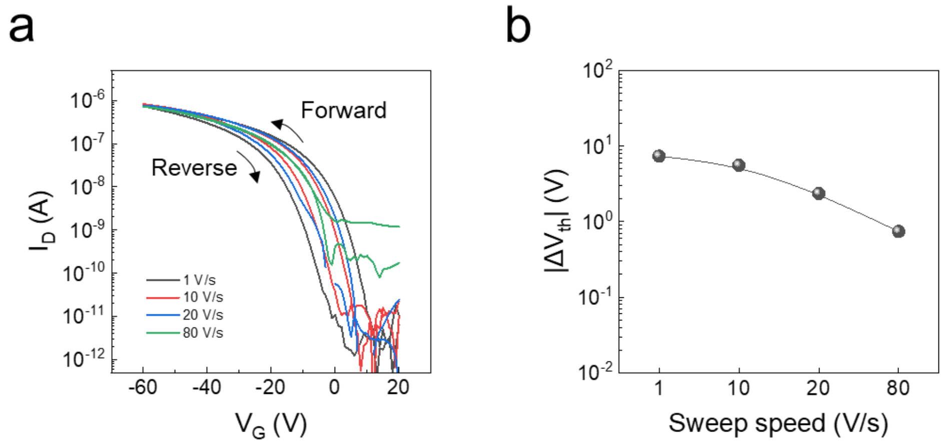

Supplementary Fig. 46. a, Transfer characteristics of the self-healing transistor and **b,** threshold voltage shift (ΔV_{th}) as a function of sweeping speed of gate voltage (V_G).

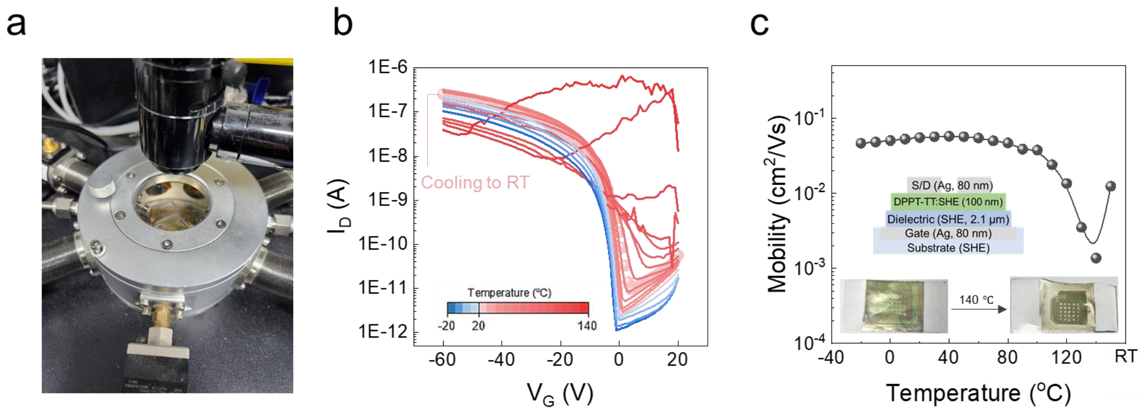

Supplementary Fig. 47. a, Photograph of temperature dependence measurement. **b,** Transfer characteristics and **c,** mobility under various temperature conditions. RT means cooling to room temperature from 140 °C. The scatter in b is the transfer curve for colling to RT condition. Inset of c is the schematic of device structure and photographs of devices before and after temperature measurement.

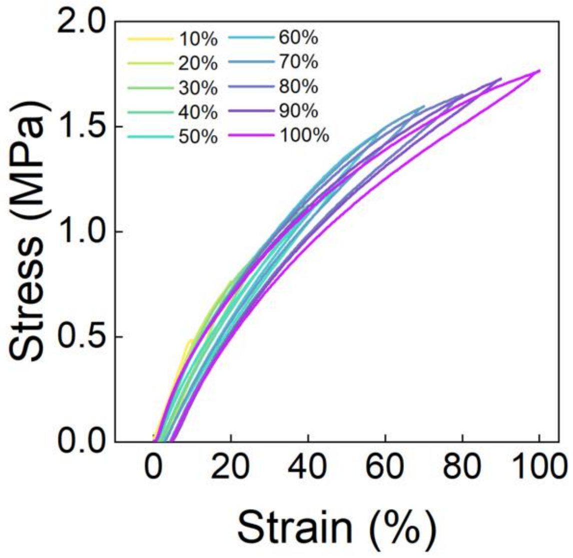

Supplementary Fig. 48. Cyclic strain-stress curves from 10% to 100% strain of fully stretchable and self-healable passive transistor array (5×5) module.

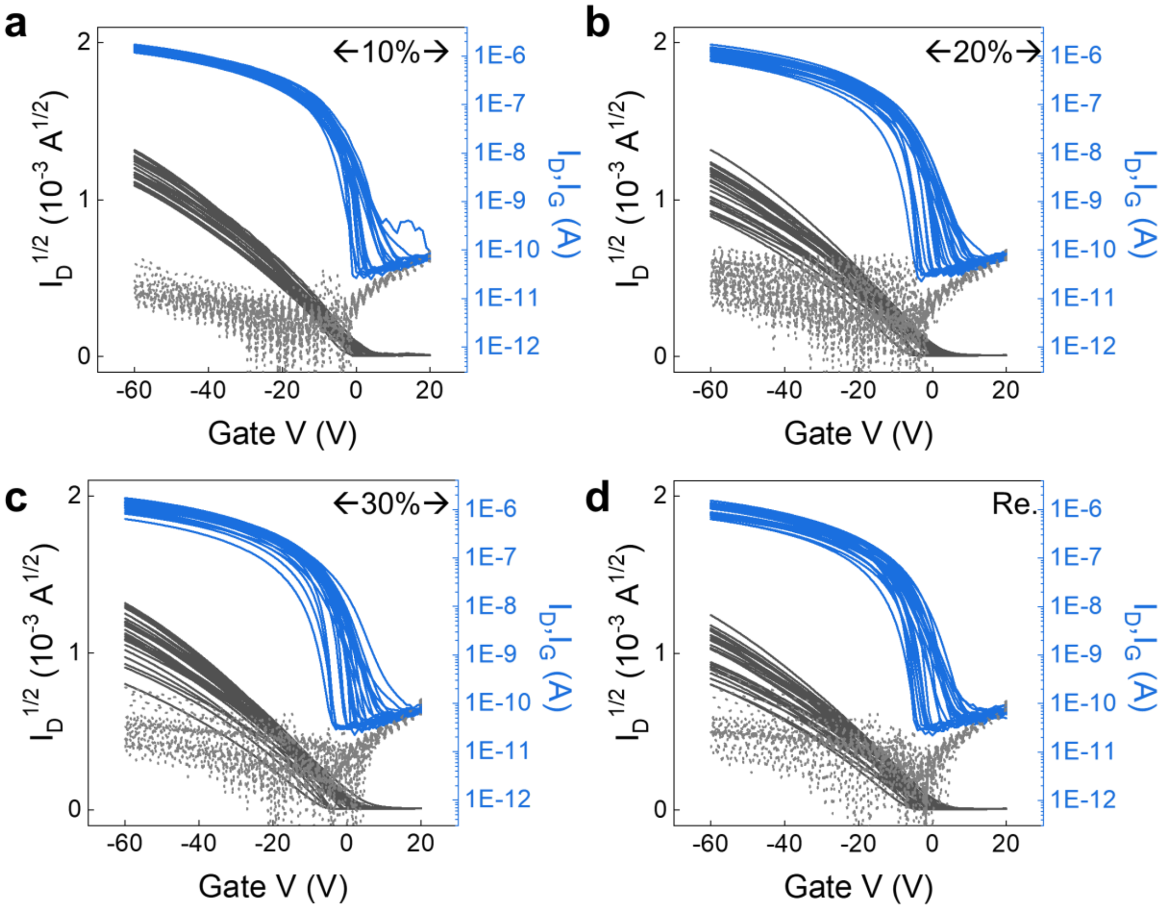

Supplementary Fig. 49. Transfer characteristics of fully stretchable and self-healable passive transistor array (5×5) under **a,** 10%, **b,** 20%, **c,** 30% and **d,** 30%-release of uniaxial strain.

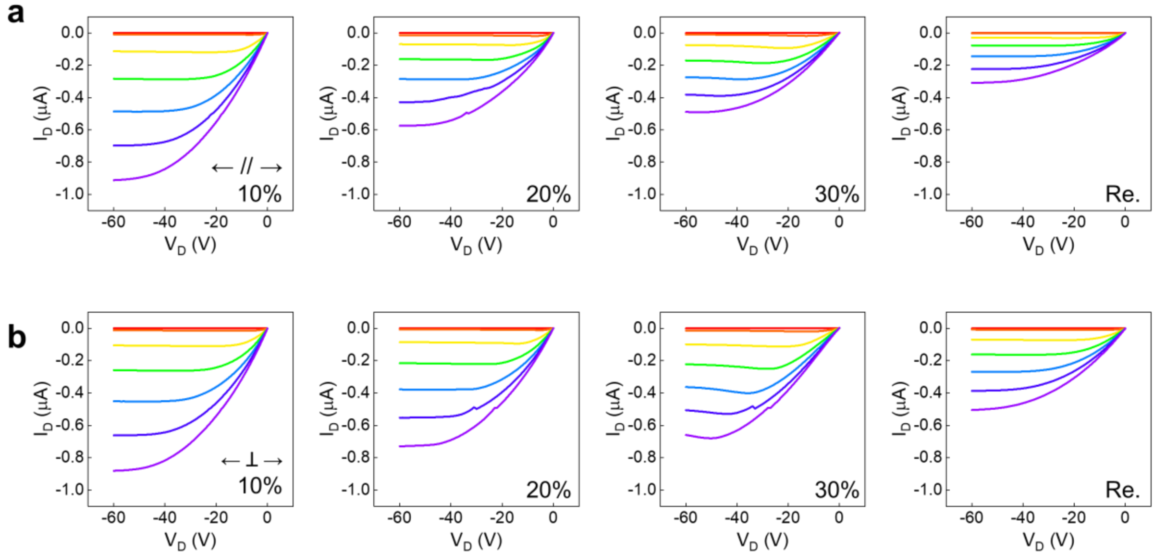

Supplementary Fig. 50. Representative output characteristics (V_G: 0 to -60 V, step: -10 V) of fully stretchable and self-healable passive arrays under uniaxial strain with **a,** parallel and **b,** perpendicular direction to channel direction.

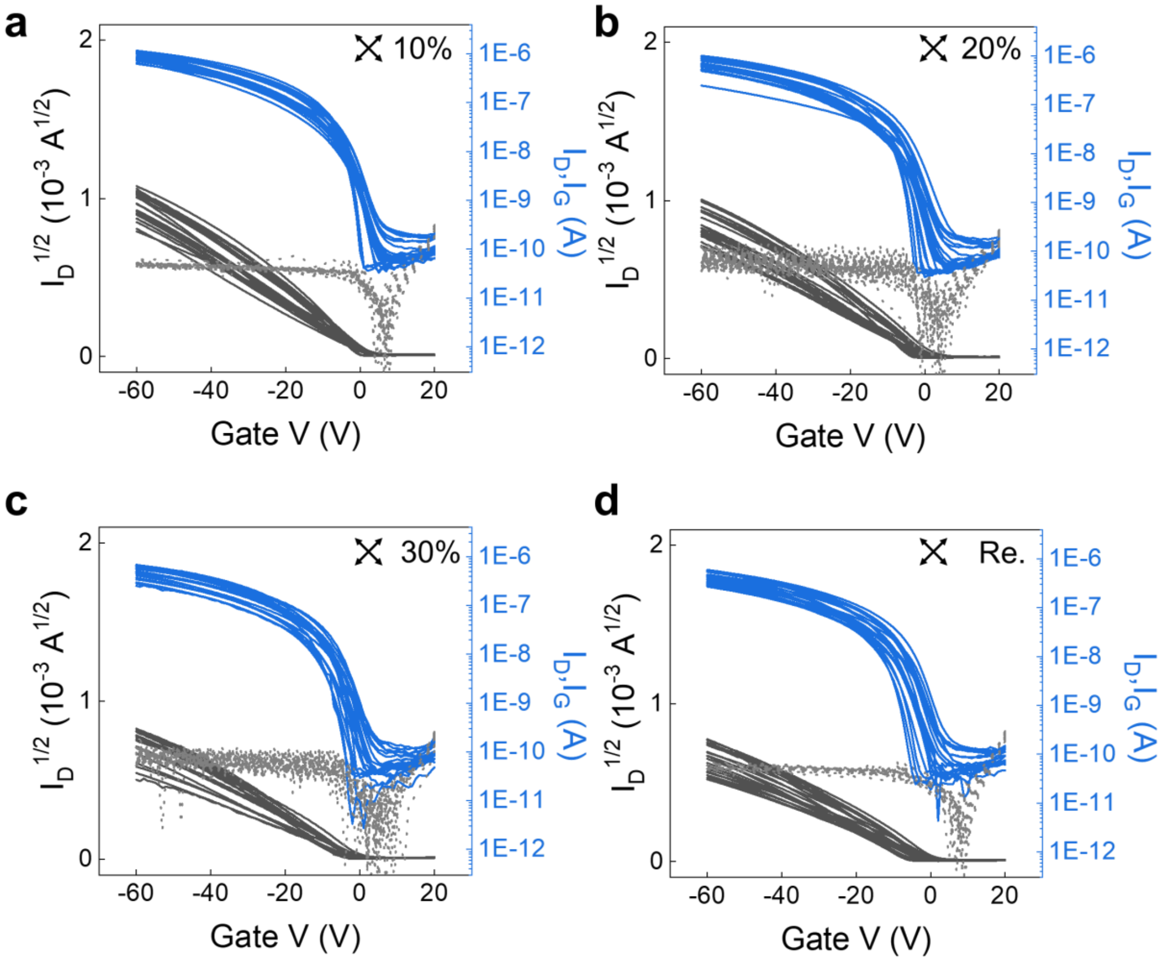

Supplementary Fig. 51. Transfer characteristics of fully stretchable and self-healable passive transistor array (5×5) under **a,** 10%, **b,** 20%, **c,** 30% and **d,** 30%-release of biaxial strain.

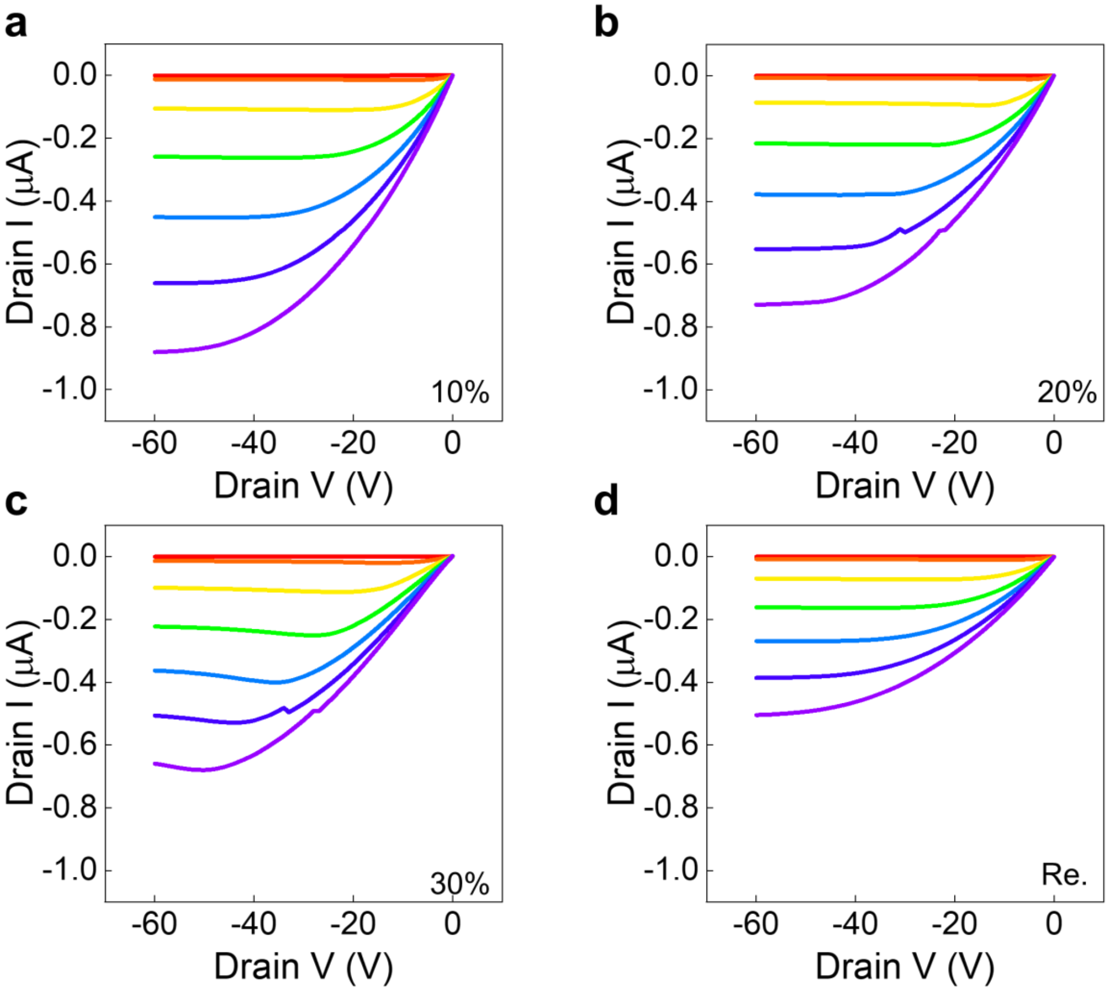

Supplementary Fig. 52. Representative output characteristics of fully stretchable and self-healable passive arrays under **a,** 10%, **b,** 20%, **c,** 30%, and **d,** 30%-release of biaxial strain.

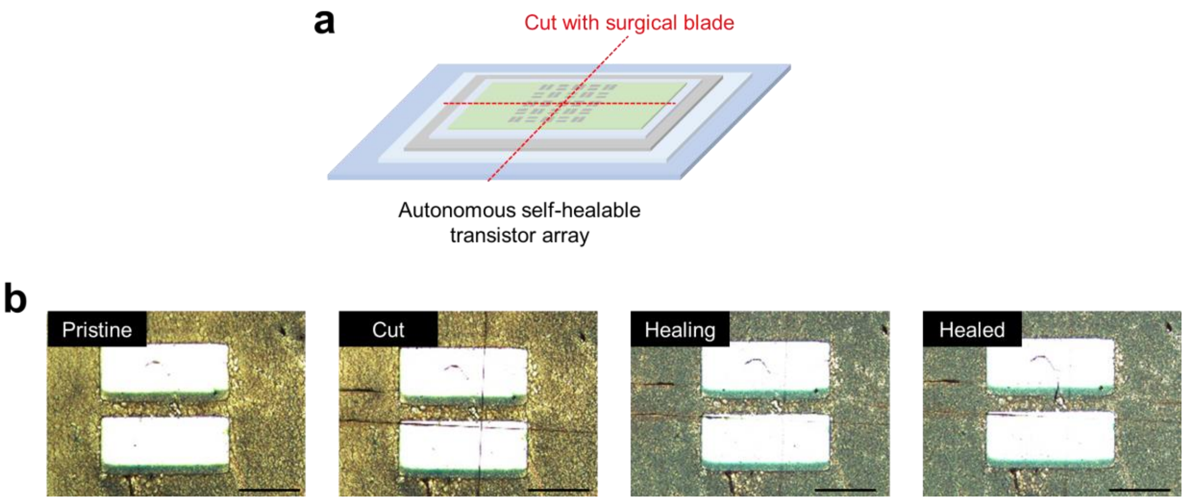

Supplementary Fig. 53. a, Schematic illustration for cross-bar shape cutting on transistor array. **b,** OM images of a unit device in center of transistor array: pristine, cut, healing (24 h) and healed (48 h) at room temperature. The scale bar is 500 μm.

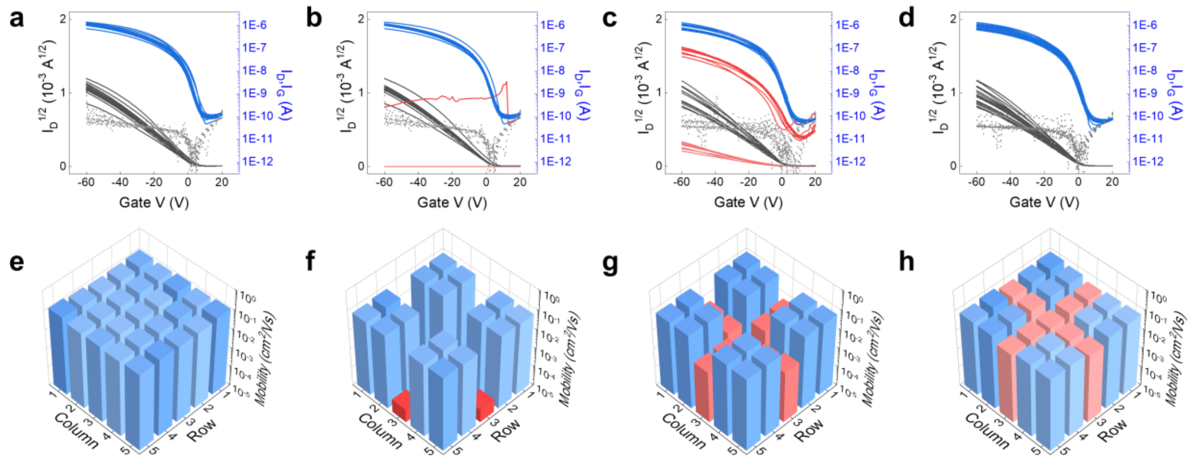

Supplementary Fig. 54. Autonomous self-healing process of 5 by 5 transistor array. Transfer characteristics of **a,** pristine, **b,** cut, **c,** healing, **d,** healed states at room temperature. Field effect mobility mapping of **e,** pristine, **f,** cut, **g,** healing (24 h), **h,** healed (48 h) states in 5 by 5 passive arrays. The cutting was conducted in a cross shape, along rows and columns, through the center.

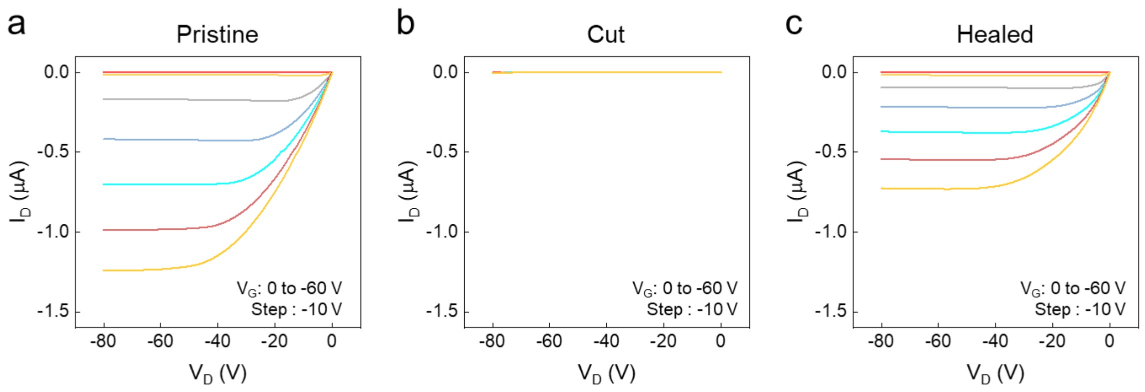

Supplementary Fig. 55. Output characteristics of **a,** pristine**, b,** cut, and **c,** healed transistors.

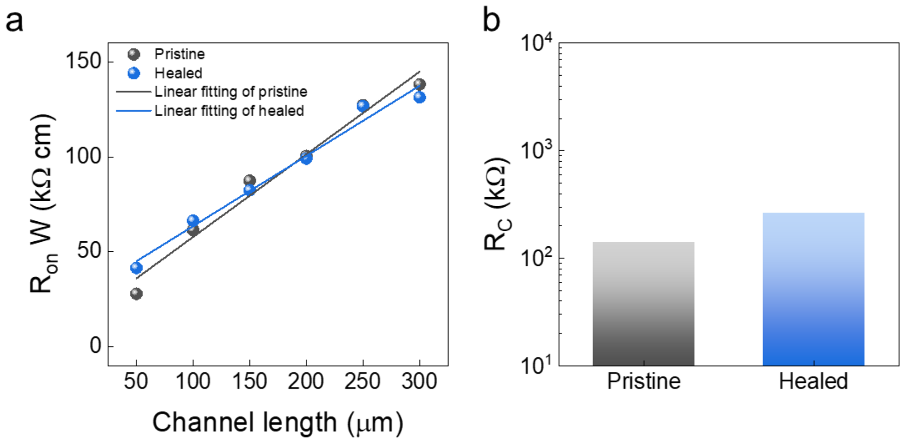

Supplementary Fig. 56. a, Resistance change of self-healing transistor as a function of channel length (V_D = -5 V). **b,** Comparison of extracted contact resistance (R_c) with pristine and healed devices.

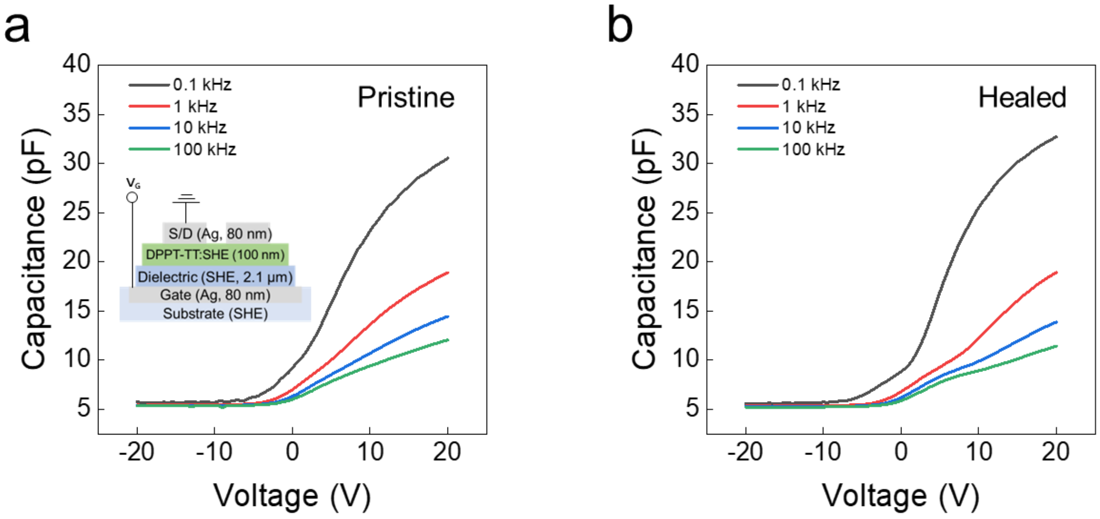

Supplementary Fig. 57. Capacitance versus voltage (C-V) curves as a function of frequency of the MIS capacitor in **a,** pristine and **b,** healed states. Inset in a is the schematic for MIS structure and the method to measure.

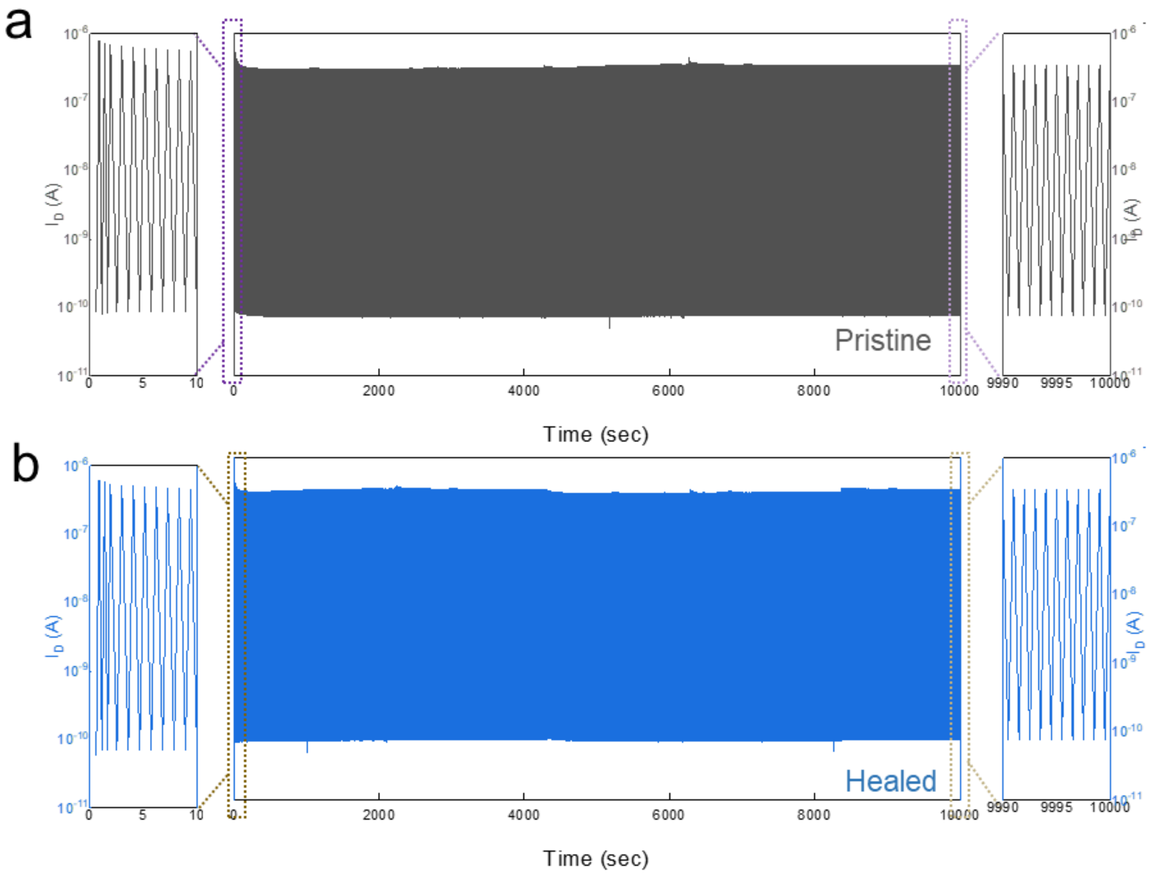

Supplementary Fig. 58. Continuous on/off switching test with 10,000 cycles of the autonomous self-healing supramolecular polymer transistor **a,** before and **b,** after healing (V_D = -10 V, and V_{GS} = -60 V_{on}, +2 V_{off}).

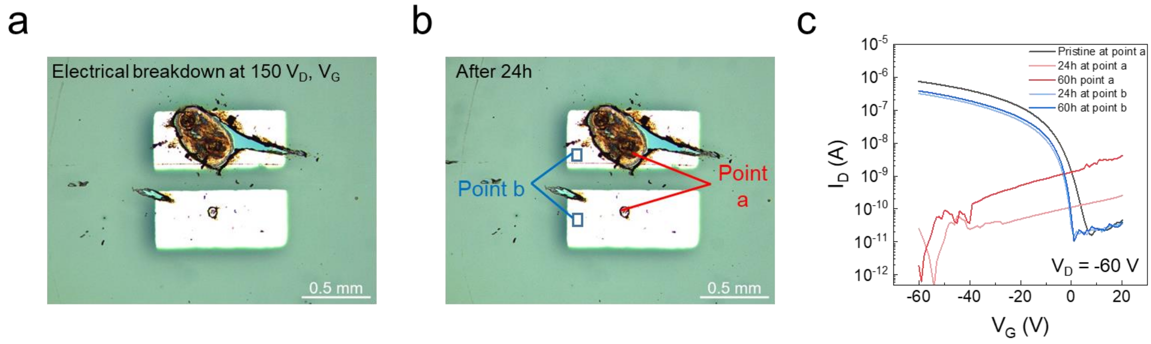

Supplementary Fig. 59. Photographs of **a,** an electrical breakdown transistor subjected to an applied voltage of -150 V_G and V_D, and **b,** the same transistor after 24 hours. **c,** I-V transfer curves of the self-healing transistor after experiencing a breakdown, 24 and 60 hours later. The transfer characteristic at point b after 24 and 60 hours.

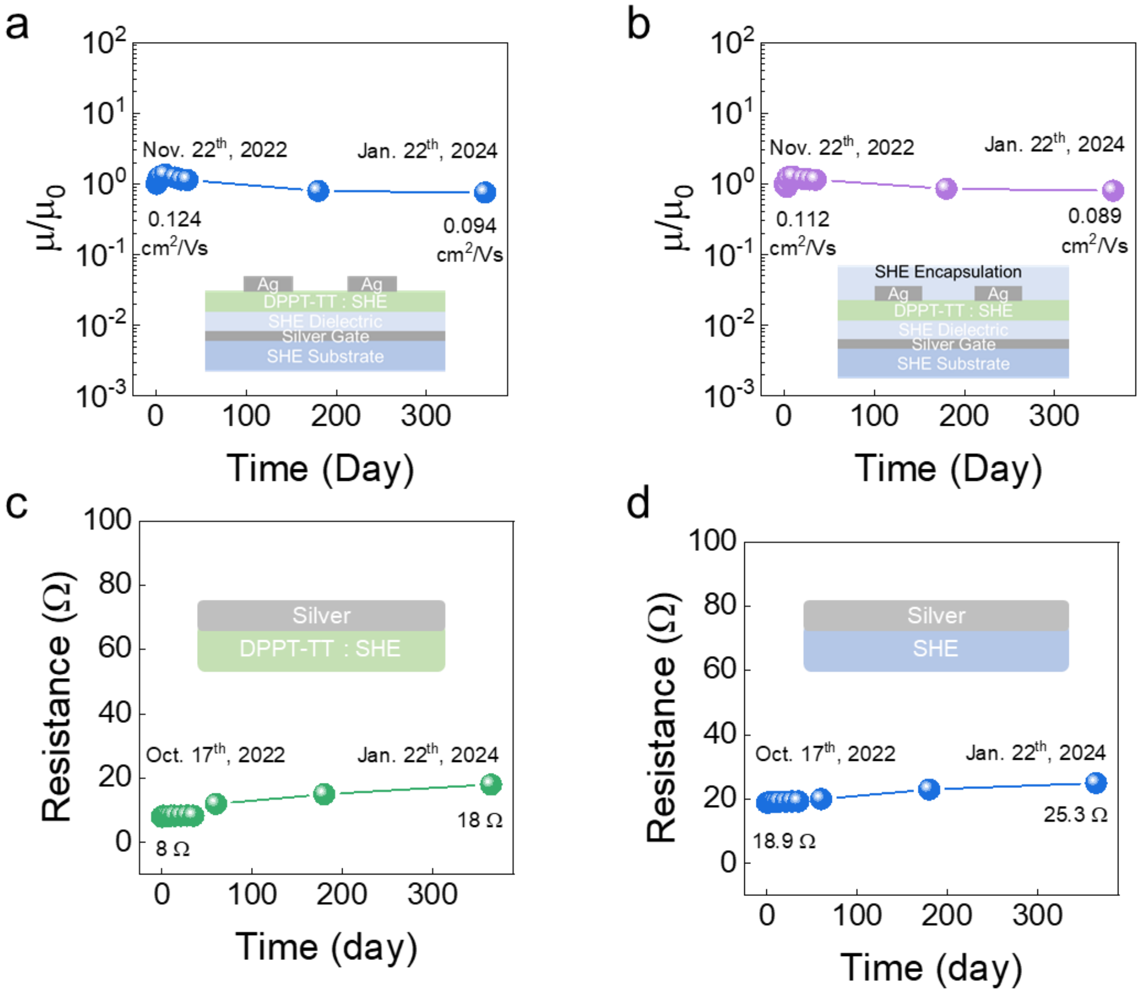

Supplementary Fig. 60. Normalized field-effect mobility changes in ambient air condition: **a,** without encapsulation and **b,** with SHE encapsulation. Resistance changes of silver electrode on **c,** semiconductor and **d,** SHE film in ambient air condition.

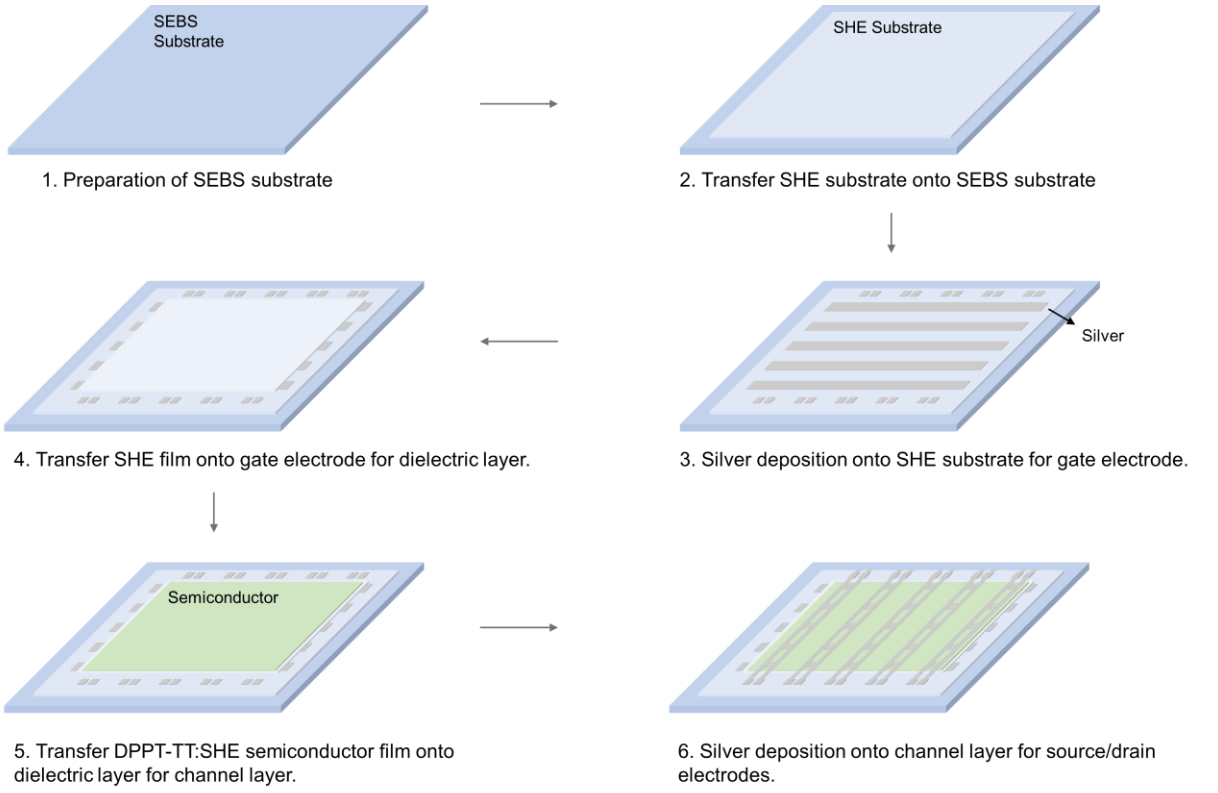

Supplementary Fig. 61. Fabrication steps for stretchable and self-healable active matrix arrays. Silver gate electrode (80 nm) was deposited onto SHE substrate (1.0 µm). SHE dielectric (2.1 µm) and semiconducting film (100 nm) on OTS-treated SiO_2/Si wafer was transferred directly onto silver electrode. Finally, silver S/D electrodes (80 nm) were deposited.

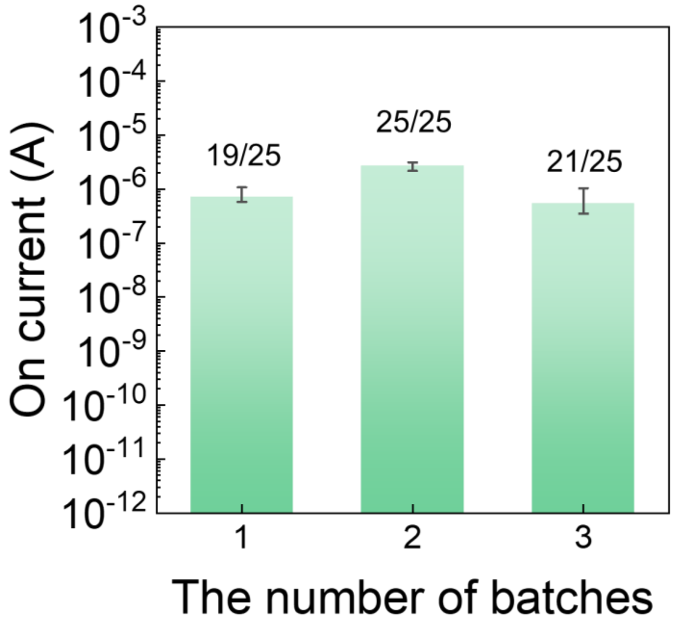

Supplementary Fig. 62. Average on-current (V_D, V_G = -60 V) of stretchable and self-healable active-matrix arrays in three different batches (x/y, x: working devices, and y: total devices). All error bars were calculated using three samples in each of three batches.

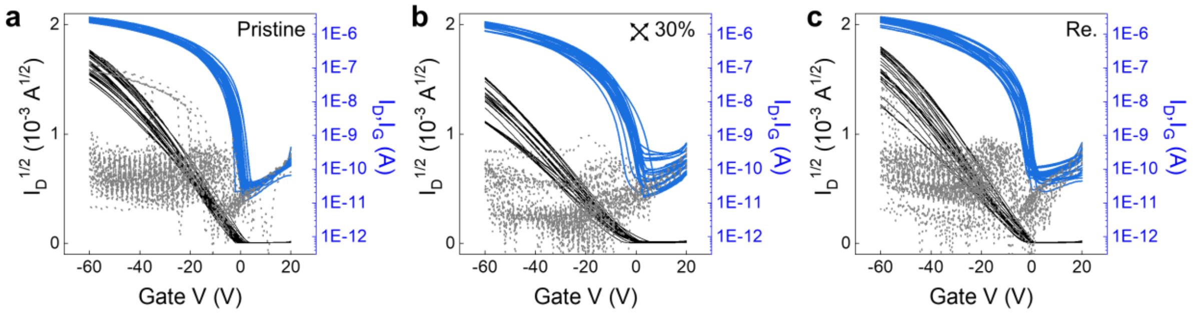

Supplementary Fig. 63. Transfer characteristics for twenty-five unit devices of stretchable and self-healable active matrix arrays under **a,** pristine, **b,** 30% biaxially stretched, **c,** 30%-released states.

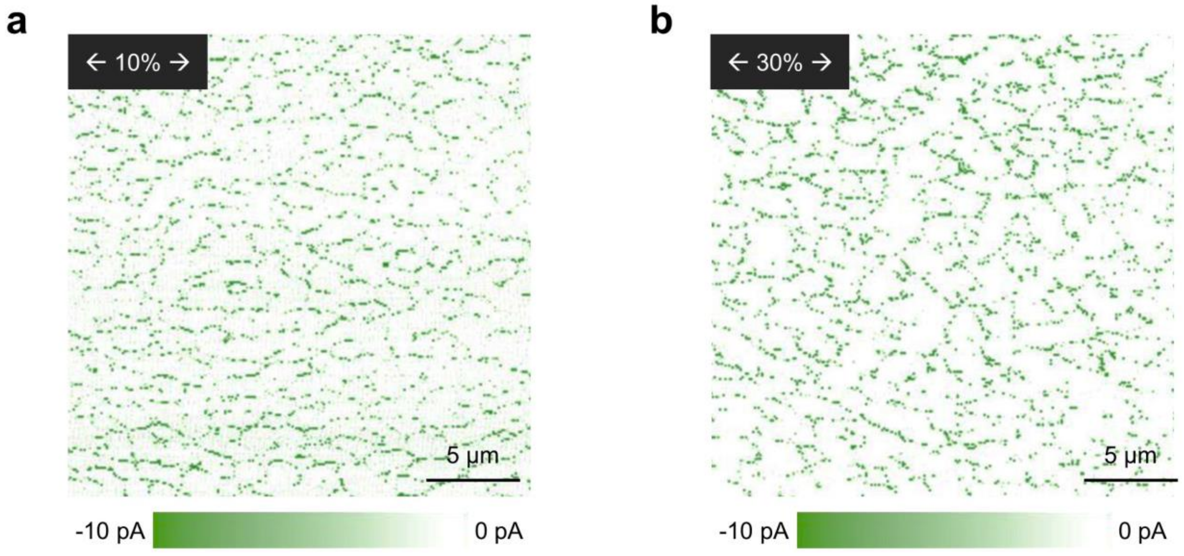

Supplementary Fig. 64. Conductive-AFM images of semiconducting films upon **a,** 10%, and **b,** 30% uniaxial strain.

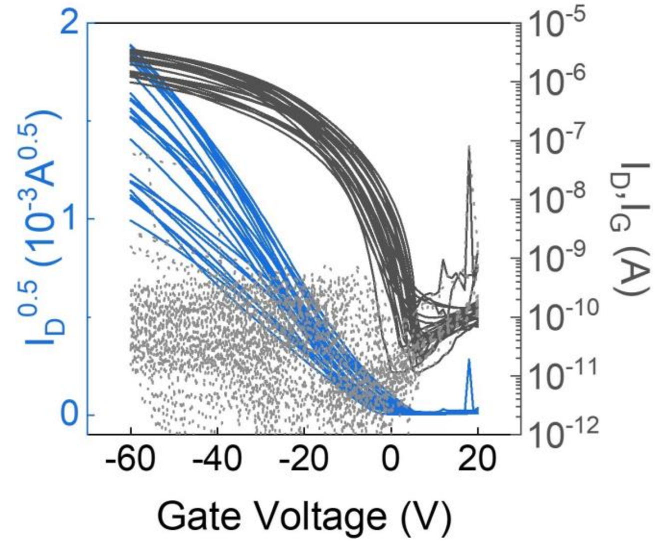

Supplementary Fig. 65. Transfer characteristics of twenty-five unit devices of active matrix arrays after healing at room temperature (48 h).

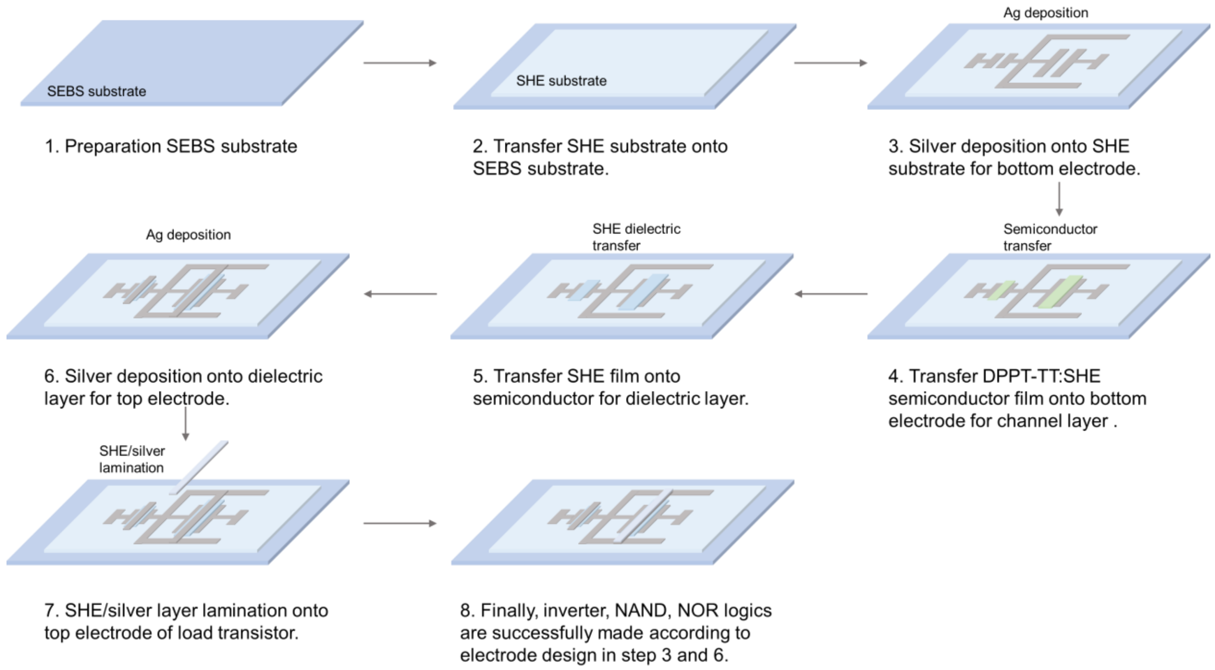

Supplementary Fig. 66. Fabrication steps for stretchable and self-healable inverter, NAND, NOR logics. Silver S/D electrode (80 nm) was deposited onto SHE substrate (1.0 µm). After deposition, semiconductor (100 nm) and SHE dielectric (2.1 µm) were directly transferred onto S/D electrode. Silver gate (80 nm) was deposited onto dielectric film, and SHE/silver film (1.0 µm/80 nm) was laminated onto gate electrode of load transistor.

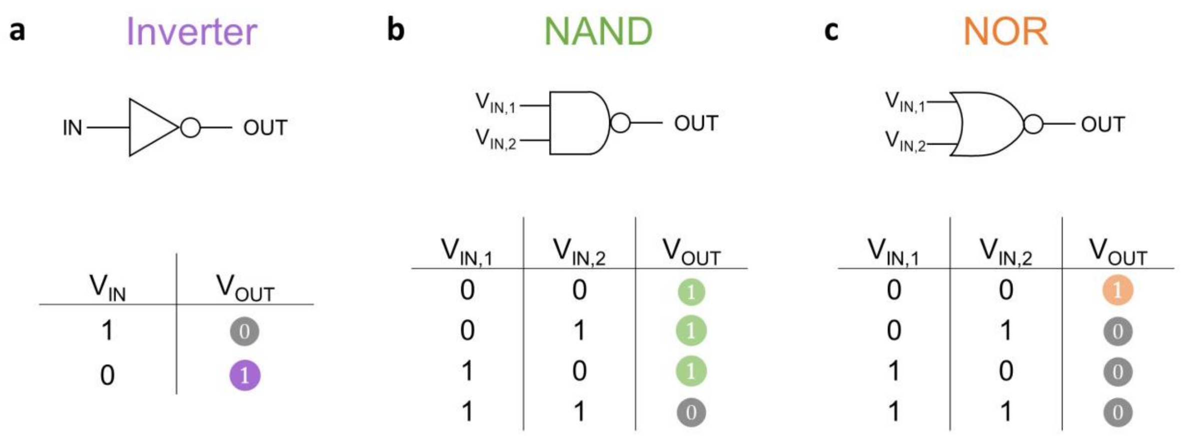

Supplementary Fig. 67. Circuit diagrams of **a,** inverter, **b,** NAND and **c,** NOR logic devices.

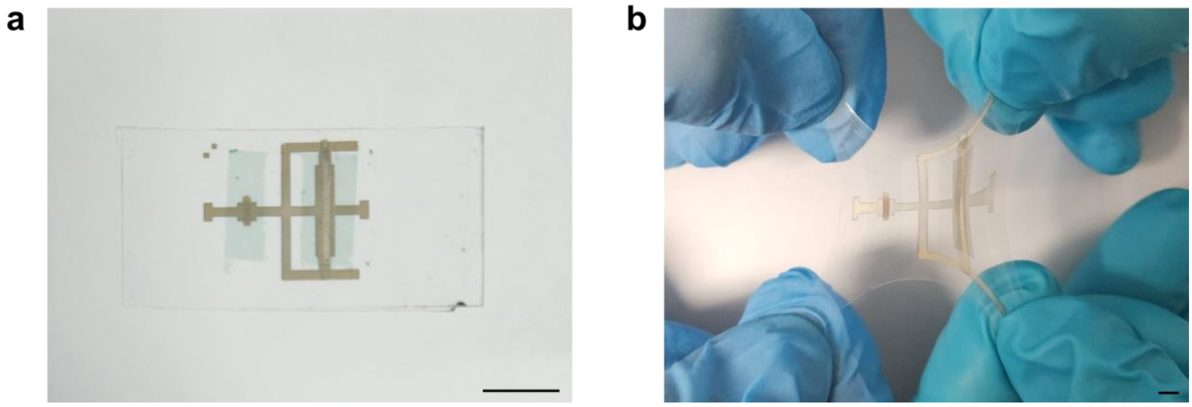

Supplementary Fig. 68. Photographs of **a,** pristine and **b,** biaxially stretched inverter devices. The scale bar is 1 cm.

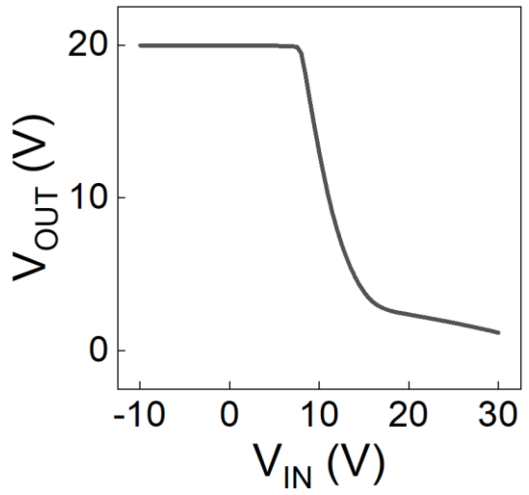

Supplementary Fig. 69. Transfer characteristic of inverter device under 10% biaxial strain at V_{DD} = 20 V.

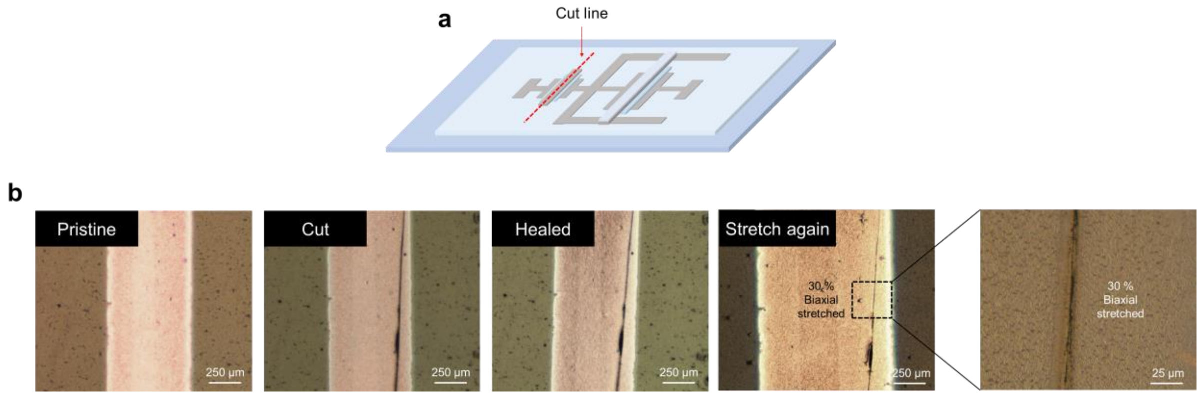

Supplementary Fig. 70. a, Schematic illustration of cutting process for inverter device. Drive transistor of device was cut using surgical blade. **b,** OM images of drive transistor under pristine, cut, healed (after 48 h) and stretched again after healed states.

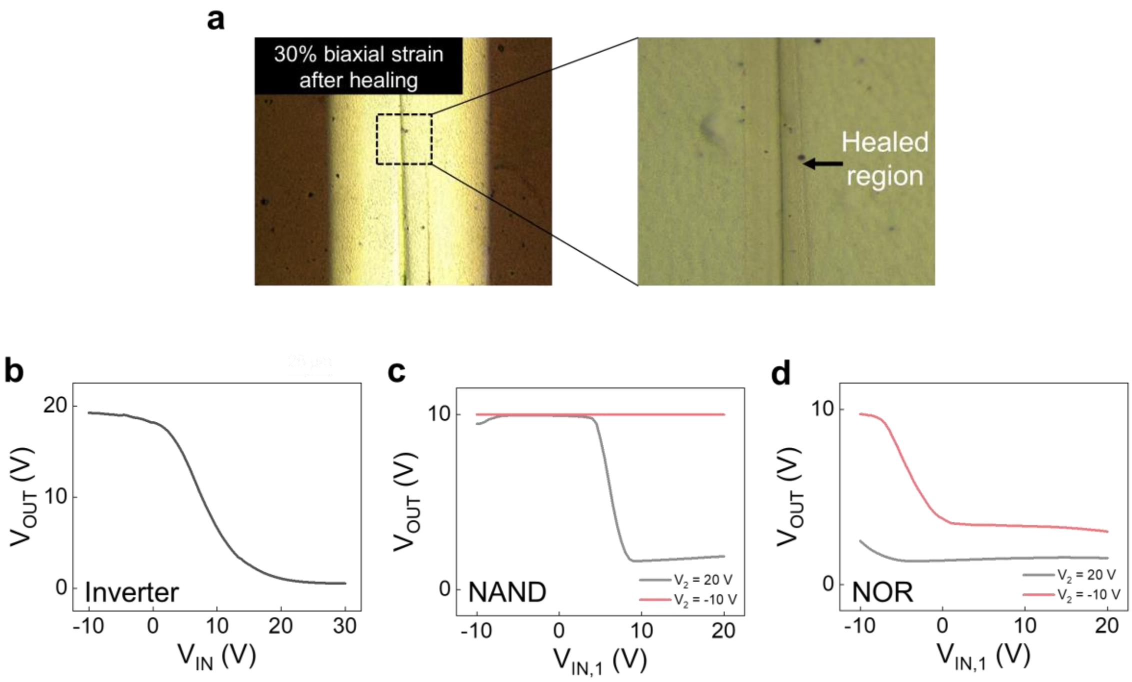

Supplementary Fig. 71. a, OM images of 30 % biaxially stretched drive transistor of healed logic device. VTCs of 30% biaxially stretched **b,** inverter, **c,** NAND and **d,** NOR logic device after healing.

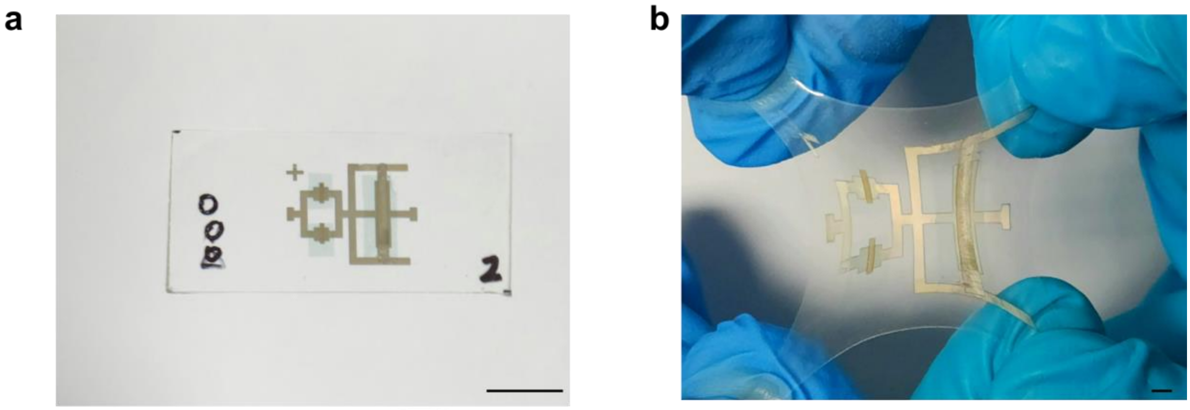

Supplementary Fig. 72. Photographs of **a,** pristine and **b,** biaxially stretched NAND devices. The scale bar is 1 cm.

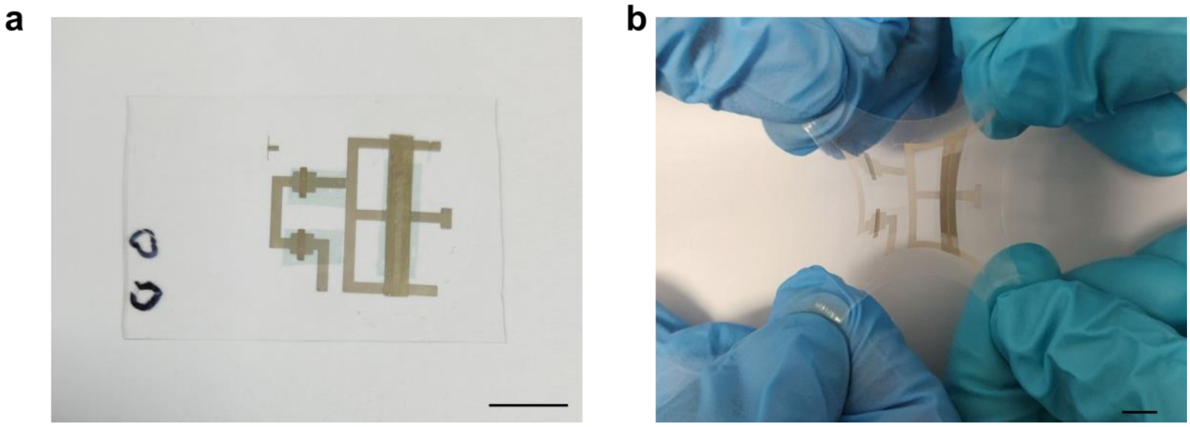

Supplementary Fig. 73. Photographs of **a,** pristine and **b,** biaxially stretched NOR devices. The scale bar is 1 cm.

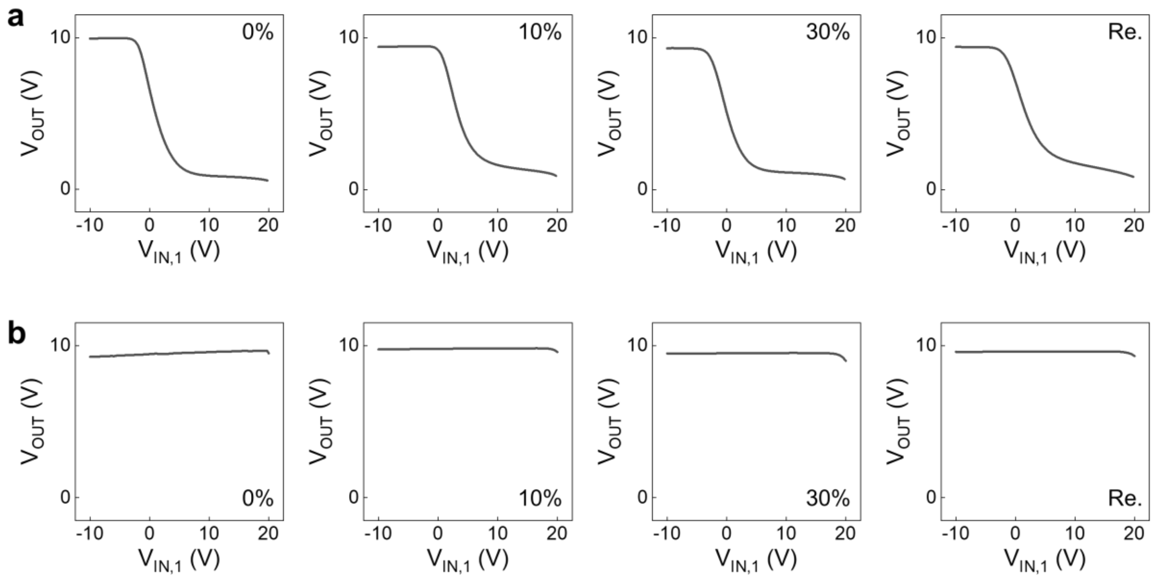

Supplementary Fig. 74. VTCs of NAND devices with various biaxial strain. Output voltages under 0 % to 30 % strain and released at **a,** $V_{IN,2}$ = 20 V and **b,** $V_{IN,2}$ = -10 V.

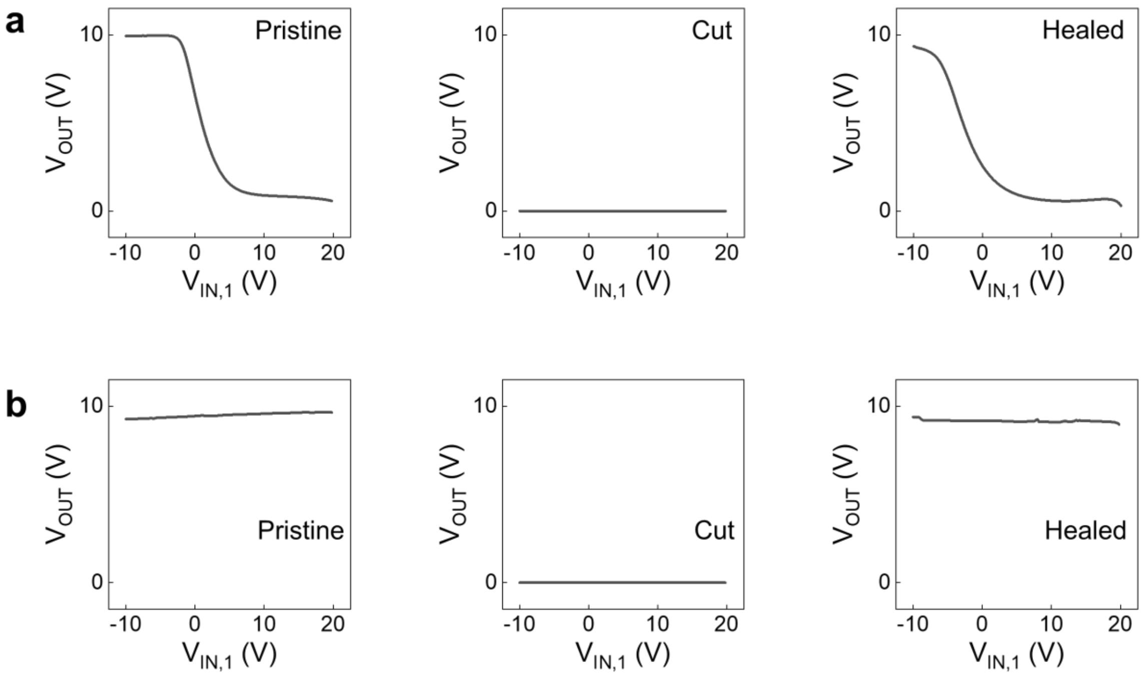

Supplementary Fig. 75. VTCs of NAND devices under autonomous self-healing process. Output voltages of pristine, cut, healed (48 h) states at **a,** $V_{IN,2}$ = 20 V and **b,** $V_{IN,2}$ = -10 V.

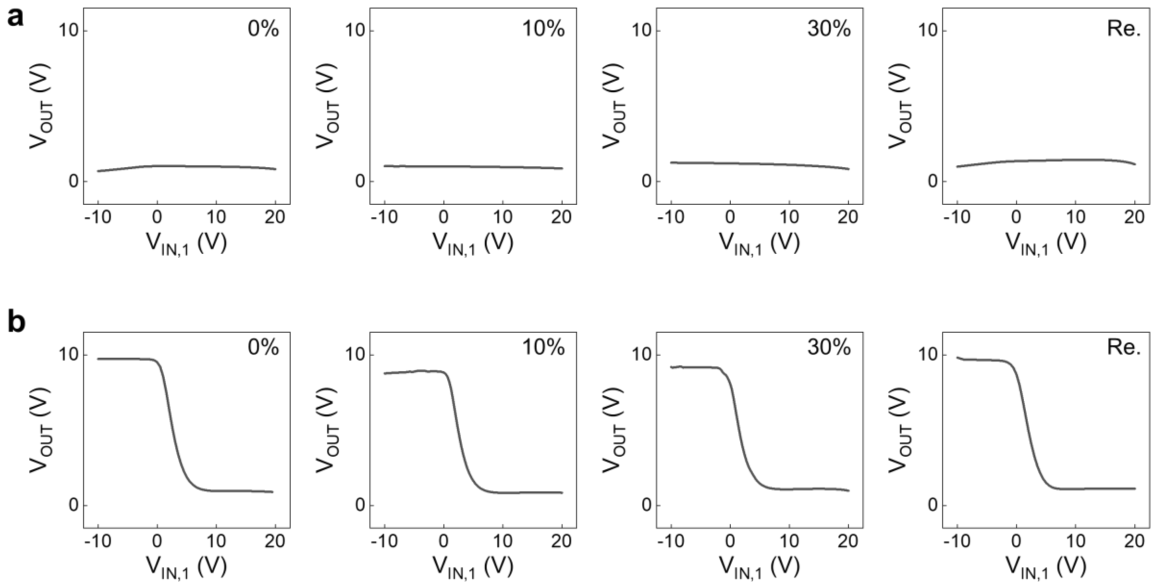

Supplementary Fig. 76. VTCs of NOR devices with various biaxial strain. Output voltages under 0 % to 30 % strain and released states at **a,** $V_{IN,2}$ = 20 V and **b,** $V_{IN,2}$ = -10 V.

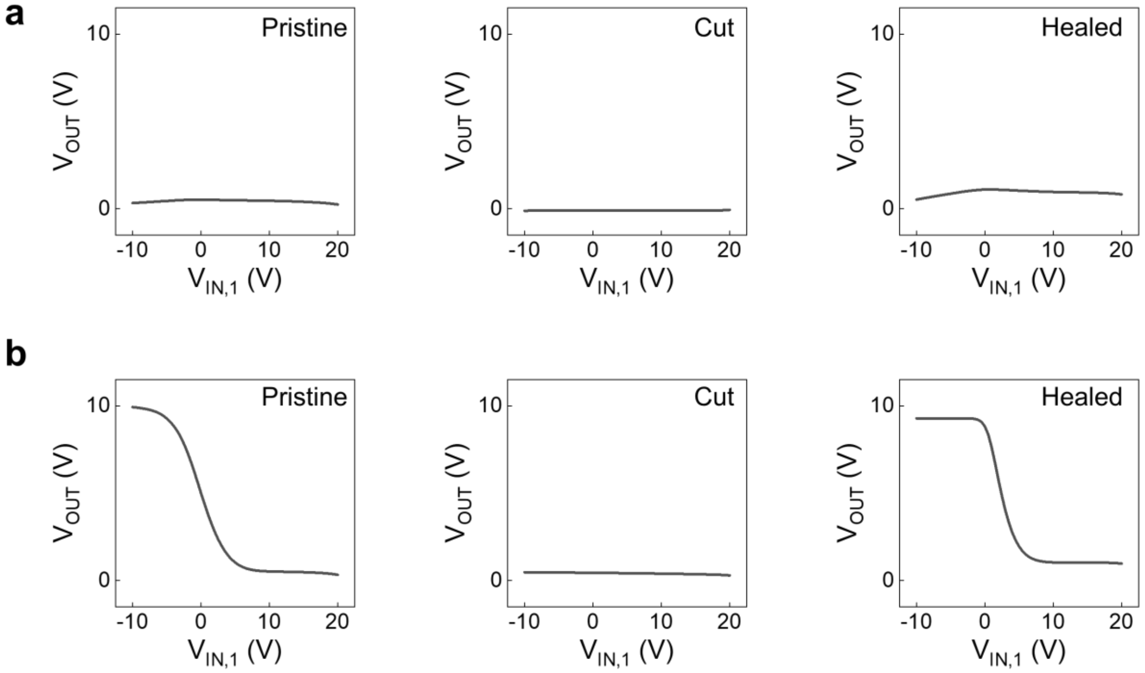

Supplementary Fig. 77. VTCs of NOR devices under autonomous self-healing process. Output voltages of pristine, cut, healed (48 h) states at **a,** $V_{IN,2}$ = 20 V and **b,** $V_{IN,2}$ = -10 V.

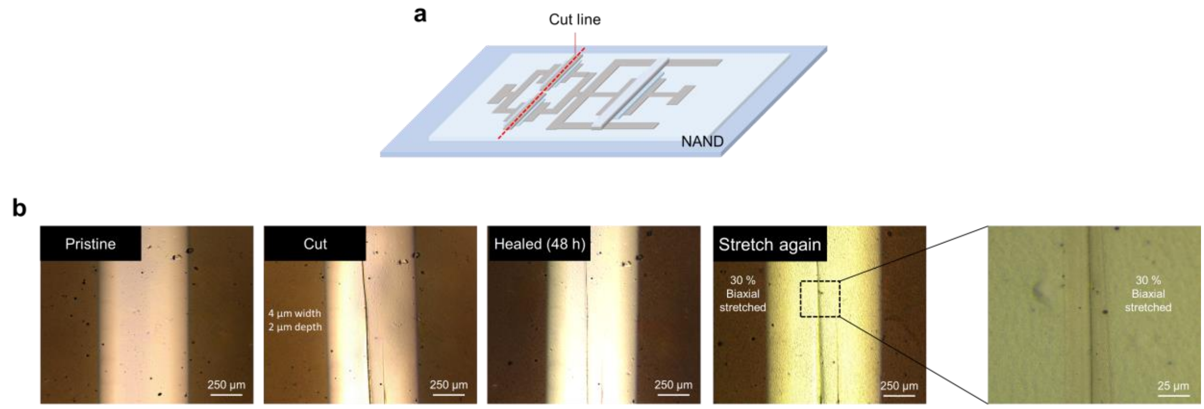

Supplementary Fig. 78. a, Schematic illustration of cutting process for NAND device. Both drive transistors of NAND were cut using surgical blade. **b,** OM images of drive transistor under pristine, cut, healed (after 48 h) states.

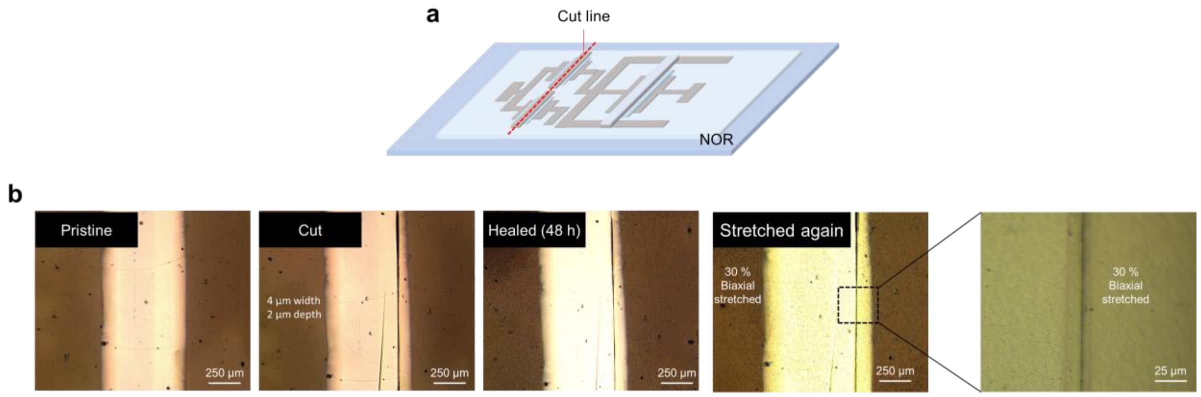

Supplementary Fig. 79. a, Schematic illustration of cutting process for NOR device. Both drive transistors of NOR were cut using surgical blade. **b,** OM images of drive transistor under pristine, cut, healed (after 48 h) and stretched again after healed states.

.

Supplementary Table 1. Device geometry and dielectric capacitance under strain.

Stretching	Strain (%)	Channel length (μm)	Channel width (μm)	Capacitance (nF/cm^2)
Uniaxial (II)	0	150	1000	1.75
	10	176	918	1.80
	20	193	883	1.88
	30	202	857	1.9
Uniaxial (=)	0	150	1000	1.75
	10	137	1180	1.85
	20	132	1180	1.85
	30	130	1257	1.93
Biaxial (×)	0	150	1000	1.75
	10	176	1032	1.86
	20	188	1092	1.94
	30	199	1148	2.24

Note that the capacitance values of gate dielectric were obtained at 1 kHz.

Supplementary Table 2. Comparison of our performance and previously reported works.

Structure	Mobility (cm^2/Vs)	Healing components	Healing method	Healing scale	Healing time	Stretchability	Ref.
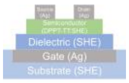	**0.11**	**All components (Source/drain, semiconductor, dielectric, gate, and substrate)**	**Autonomous healing**	**5 μm**	**36 hours**	**Uniaxial: 30%<br>Biaxial: 30%**	**This work**
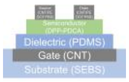	0.286	Semiconductor	Solvent vapor and heat treatments	0.05 μm	N/A	Uniaxial: 100%<br>Biaxial: N/A	#4
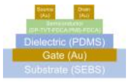	0.076	Semiconductor	Autonomous healing	0.2 μm	24 hours	Uniaxial: 100%<br>Biaxial: N/A	#5
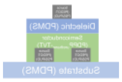	0.002	Semiconductor	Solvent vapor and heat treatments	0.05 μm	N/A	Uniaxial: 20%<br>Biaxial: N/A	#6
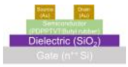	0.16	Semiconductor	Autonomous healing	N/A	Few seconds	Only semiconductor<br>Uniaxial: 150%<br>Biaxial: N/A	#7
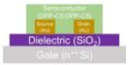	0.4	Semiconductor	Melt process	20 μm	N/A	Non-stretchable	#8

Reference

1. Stafford, C. M. et al. A buckling-based metrology for measuring the elastic moduli of polymeric thin films. *Nat. Mater.* **3**, 545-550 (2004).
2. Owens, D. K. et al. Estimation of the surface free energy of polymers. *J. Appl. Polym. Sci.* **13**, 1741–1747 (1969).
3. Kim, M. H. et al. Mechanically robust stretchable semiconductor metallization for skin-inspired organic transistors. *Sci. Adv.* **8**, eade2988 (2022).
4. Oh, J. Y. et al. Intrinsically stretchable and healable semiconducting polymer for organic transistors. *Nature* **539**, 411-415 (2016).
5. Oh, J. Y. et al. Stretchable self-healable semiconducting polymer film for active-matrix strain-sensing array. *Sci. Adv.* **5**, eaav3097 (2019).
6. Lee, M. Y. et al. Regular H-Bonding-Containing polymers with stretchability up to 100% external strain for self-healable plastic transistors. *Chem. Mater.* **32**, 1914-1924 (2020).
7. Zhang, S. et al. Tacky elastomers to enable tear-resistant and autonomous self-healing semiconductor composites. *Adv. Funct. Mater.* **30**, 2000663 (2020).
8. Zhao, Y. et al. Melt-processing of complementary semiconducting polymer blends for high performance organic transistors. *Adv. Mater.* **29**, 1605056 (2017).
